# Neuroinflammation-Induced Upregulation of Glial Cathepsin X Expression and Activity *in vivo*

**DOI:** 10.3389/fnmol.2020.575453

**Published:** 2020-11-20

**Authors:** Anja Pišlar, Larisa Tratnjek, Gordana Glavan, Nace Zidar, Marko Živin, Janko Kos

**Affiliations:** ^1^Department of Pharmaceutical Biology, Faculty of Pharmacy, University of Ljubljana, Ljubljana, Slovenia; ^2^Institute of Pathophysiology, Medical Faculty, University of Ljubljana, Ljubljana, Slovenia; ^3^Institute of Cell Biology, Medical Faculty, University of Ljubljana, Ljubljana, Slovenia; ^4^Department of Biology, Biotechnical Faculty, University of Ljubljana, Ljubljana, Slovenia; ^5^Department of Pharmaceutical Chemistry, Faculty of Pharmacy, University of Ljubljana, Ljubljana, Slovenia; ^6^Department of Biotechnology, Jožef Stefan Institute, Ljubljana, Slovenia

**Keywords:** neuroinflammation, neurodegeneration, lipopolysaccharide, glia, cathepsin X, neuroprotection

## Abstract

Neuroinflammation is an important factor in the pathogenesis of neurodegenerative diseases. Microglia-derived lysosomal cathepsins have been increasingly recognized as important inflammatory mediators that trigger signaling pathways that aggravate neuroinflammation. *In vitro*, a contribution to neuroinflammation processes has been shown for cathepsin X: however, the expression patterns and functional role of cathepsin X in neuroinflammatory brain pathology remain elusive. In this study we analyzed the expression, activity, regional distribution and cellular localization of cathepsin X in the rat brain with neuroinflammation-induced neurodegeneration. The unilateral injection of lipopolysaccharide (LPS) induced a strong upregulation of cathepsin X expression and its activity in the ipsilateral striatum. In addition to the striatum, cathepsin X overexpression was detected in other brain areas such as the cerebral cortex, corpus callosum, subventricular zone and external globus pallidus, whereas the upregulation was mainly restricted to activated microglia and reactive astrocytes. Continuous administration of the cathepsin X inhibitor AMS36 indicated protective effects against LPS-induced striatal degeneration, as seen by the attenuated LPS-mediated dilation of the lateral ventricles and partial decreased extent of striatal lesion. Taken together, our results indicate that cathepsin X plays a role as a pathogenic factor in neuroinflammation-induced neurodegeneration and represents a potential therapeutic target for neurodegenerative diseases associated with neuroinflammation.

## Introduction

Neuroinflammation is associated with many neurodegenerative diseases, including Parkinson's disease (PD), Alzheimer's disease (AD), Huntington disease, amyotrophic lateral sclerosis and multiple sclerosis. Accumulating evidence suggests that chronic innate neuroinflammation mediated by microglia and astrocytes is implicated in the progressive nature of these neurodegenerative disorders. Whereas in healthy conditions microglia display key beneficial functions that are crucial for maintaining central nervous system (CNS) homeostasis, their chronic activation in neurodegeneration results in their altered functionality, which is thought to ultimately play an instrumental and detrimental role in disease pathogenesis (Dorothee, 2018). For example, array of evidence generated by animal experiments and clinical studies indicates that neuroinflammatory processes are crucial for the initiation and progression of PD (McGeer et al., 2005; McGeer and McGeer, 2008; Wang et al., 2015), which is characterized by a progressive degeneration of the dopaminergic projection between the substantia nigra *compacta* (SNc) and the striatum, followed by a decrease in striatal dopamine (DA) levels (Hornykiewicz, 1993; Youdim and Riederer, 1997). During neuroinflammation, activated microglia and astrocytes release a variety of cytokines, chemokines and toxic factors, all of which may lead to subsequent neuronal toxicity and aggressive neuronal loss that drives the pathogenic progress related to PD (Choi et al., 2009; Menza et al., 2010; More et al., 2013; Niranjan, 2014). Similarly, increasing evidence suggests that AD pathogenesis strongly interacts with neuroinflammation (reviewed in Heneka et al., 2015). Misfolded and aggregated proteins bind to pattern recognition receptors on glia cells and trigger an innate immune response, characterized by the release of inflammatory mediators, which contribute to the pathogenesis of AD. On the other hand, activated microglia have been linked with neuroprotection in several studies, and it has been suggested that glial cells are activated in response to neuronal injury, with subsequent release of neurotrophic factors (Mallat et al., 1989; Lindsay et al., 1994; Elkabes et al., 1996; Nakajima et al., 2001). Nevertheless, the characterization of the endogenous biomolecules involved in neuroinflammation-induced neurodegeneration may be critical in the development of novel therapeutic strategies for the treatment of diseases such as PD and AD, since effective treatments are lacking. Indeed, some studies have shown beneficial and promising effects of anti-inflammatory agents, however, mixed results and inconsistencies between the preclinical and clinical studies require a better understanding of neuroinflammatory processes in neurodegenerative diseases (Schwartz and Shechter, 2010; Wang et al., 2015).

Increasing evidence shows that activated microglia induces the expression and secretion of lysosomal cathepsins, in particular during the early stage of neuroinflammation, to trigger signaling cascades that aggravate neurodegeneration (Kingham and Pocock, 2001; Wendt et al., 2009; Terada et al., 2010; Clark and Malcangio, 2012; Fan et al., 2012, 2015; Hafner et al., 2013; Pislar et al., 2017). To date, most neuroinflammation research has focused on cysteine cathepsins, which are the largest cathepsin family, comprised of 11 members (cathepsins B, C, F, H, K, L, O, S, W, V, and X). These cathepsins possess a conserved active site involving cysteine, histidine and aspargine residues in a catalytic triad (Rawlings et al., 2012). They are primarily responsible for terminal protein degradation in lysosomes; however, their role in regulating a number of other important physiological and pathological processes has been demonstrated (Obermajer et al., 2006; Pislar and Kos, 2014; Pislar et al., 2015; Stoka et al., 2016).

The role of cathepsin X, a cysteine cathepsin with solely carboxypeptidase activity, has been chiefly restricted to cells of the immune system, predominantly monocytes, macrophages and dendritic cells (Kos et al., 2005, 2009). Cathepsin X expression and proteolytic activity have also been found to be strongly upregulated in the mouse brain, with a preference for glial cells and aged neurons (Stichel and Luebbert, 2007; Wendt et al., 2007). In a transgenic mouse model of AD, cathepsin X upregulation in microglial cells surrounding amyloid plaques has been observed, and further, the involvement of cathepsin X in amyloid-β-related neurodegeneration through proteolytic cleavage of the C-terminal end of γ-enolase has been suggested (Wendt et al., 2007; Hafner et al., 2013; Pislar and Kos, 2013). Additionally, cathepsin X also promotes the apoptosis of neuronal cells induced by the neurotoxin 6-hydroxydopamine (6-OHDA), which was reversed with cathepsin X downregulation or inhibition by the specific cathepsin X inhibitor (Pislar et al., 2014). Moreover, *ex vivo* cathepsin X expression and its activity have been found to be strongly increased in the SNc of hemi-parkinsonian rats with 6-OHDA-induced excitotoxicity in the unilateral medial forebrain bundle, indicating that cathepsin X may be involved in the pathogenic cascade event in PD (Pislar et al., 2018). In addition to a role in neurodegeneration, the involvement of cathepsin X in inflammation-induced neurodegeneration has been reported. Substantially increased secretion of cathepsin X from microglia has been observed in response to the inflammatory stimulus induced by lipopolysaccharide (LPS), leading to microglia activation-mediated neurodegeneration (Wendt et al., 2009; Pislar et al., 2017). To date, the cathepsin X expression and its role in inflammation-induced neurodegeneration have been studied mainly *in vitro*; however, the expression pattern and cellular localization of cathepsin X in the brain, as well as the functional role of cathepsin X in chronic inflammation related to neurodegeneration still remain unclear.

Animal models have become essential to study the involvement of neuroinflammatory processes in the development of neurodegenerative diseases. The current *in vivo* study explored the expression pattern and cellular localization of cysteine peptidase cathepsin X in the rat brain following unilateral striatal LPS administration, which induces inflammation by activation of glial cells that release several neurotoxins such as inflammatory cytokines, N-methyl- D-aspartate, receptor agonists, oxygen-free radicals, and nitric oxide (Espinosa-Oliva et al., 2013). We have demonstrated here LPS-induced cathepsin X overexpression restricted to microglia cells and reactive astrocytes in the ipsilateral striatum as well as brain areas such as the ipsilateral cortex, corpus callosum and external globus pallidus. To gain further insight into the role of upregulated cathepsin X in neuroinflammation-induced neurodegeneration, LPS injected rats were treated with cathepsin X-specific inhibitor AMS36, which illustrated the potential to protect against LPS-induced neuroinflammation.

## Materials and Methods

### Animals

Male Wistar rats from the Medical Experimental Center, Medical Faculty, University of Ljubljana, Slovenia, weighing 200–250 g at the beginning of the experiments. The animals were kept under standard laboratory conditions (22 ± 1°C; 70 ± 10% RH; 12 h light/dark cycle; 2-3 rats per cage) with free access to food and water. All animal-related procedures were conducted in accordance with the European Communities Council Directive of 2010 (2010/63/UE) and the National Veterinary Institute Guide for the Care and Use of Laboratory Animals. Care was taken to minimize the number of experimental animals and their suffering. The experiments were approved by the Administration of the Republic of Slovenia for Food Safety, Veterinary Sector and Plant Protection (Ref. No. 34401-48/2012/5). The total number of animals used was 11, divided into two groups: DMSO/saline treated LPS-hemi-leisoned rats (*n* = 5, group LPS) and irreversible inhibitor AMS36 treated LPS-hemi-lesioned rats (*n* = 6, group LPS/AMS36) The animals were kept in IVC (Individually ventilated cage), two animals in each cage.

### Unilateral Intrastriatal Lipopolysaccharide-Induced Lesion

Surgical procedures for intrastriatal LPS injection were adopted from Choi et al. (2009). For the unilateral intrastriatal injection of LPS (Salmonella Minnesota; Sigma Aldrich, St. Louis, MO, United States), male Wistar rats were deeply anesthetized with sodium phenobarbital (Sigma-Aldrich; 50 mg/kg i.p.) and then positioned in a stereotaxic frame (TrentWells, South Gate, CA, United States) with the incisor bar at the level of the ear. LPS dissolved in saline (2.5 μg/μL) was injected into the right striatum (3 μL/site) at the following coordinates (in mm): site 1, anteroposterior (AP) 1.0, mediolateral (ML) 2.0, dorsoventral (DV) −4.5; site 2, AP 1.0, ML 3.0, DV −5.5; site 3, AP −0.5, ML 3.0, DV −4.5; site 4, AP −0.5, ML 4.0, DV −6.0. Saline was injected into the left striatum with parallel coordinates. The cannula was left at the injection site for an additional 3 min post-injection before being slowly retracted. After surgery, animals were kept on a heating pad until recovery from surgery and subcutaneous saline was given for aid in postoperative recovery. The unilateral LPS model provides self-control for intervention.

In the preliminary study, two animals were sacrified 4 weeks after the unilateral intrastriatal LPS injection. Brain tissue from this group of animals was examined for cathepsin X expression by immunohistochemistry and cathepsin X activity was determined. Increased cathepsin X expression and activity were demonstrated in the LPS-injected striatum (Supplementary Figure 1).

In order to investigate the effect of cathepsin X inhibition on the LPS-induced lesion, a specific, irreversible inhibitor of cathepsin X AMS36, synthesized as reported (Sadaghiani et al., 2007; Pislar et al., 2014), was used. The potency of cathepsin X inhibitor has been determined in the preliminary study, where male Wistar rats were randomly allocated in four groups, treated either with dimethyl sulfoxide (DMSO)/saline solution ratio 1:10 (vol/vol) (i.p) or AMS36 dissovled in DMSO/saline solution ratio 1:10 (vol/vol) at a dose of 50 mg/kg (i.p.) and scarified at day 1 (*n* = 4, group DMSO/saline; *n* = 4, group AMS36) and day 2 (*n* = 4, group DMSO/saline; *n* = 4, group AMS36) for protein extraction from cerebellum to determine cathepsin X activity. Based on the obtained cathepsin X activity presented in the Supplementary Figure 2, we treated the male Wistar rats with vehicle or AMS36 in the subsequent experiments 2 days prior unilateral LPS injection and then every fourth day for 4 weeks.

For the experiments, the male Wistar rats were randomly allocated in two groups, both receiving unilateral intrastriatal injection of LPS into the right striatum. No exclusion criteria were predetermined and no blinding procedure was performed. The first group of animals was treated with DMSO/saline solution, whereas the second group was treated with cathepsin X inhibitor AMS36. Namely, the first group (*n* = 5, group LPS) of animals was treated with DMSO/saline solution ratio 1:10 (vol/vol) (i.p.) 2 days prior to LPS/saline injection (into the right and left striatum, respectively) and then every fourth day with DSMO/saline (i.p.) for 4 weeks. After 4 weeks, the animals were sacrificed, and brain tissue from this group of animals was further examined for model evaluation and cathepsin X expression analysis by histological staining, immunohistochemistry and immunofluorescence labeling and protein extraction to determine cathepsin X protein level expression and activity and tyrosine hydroxylase (TH) and inducible nitric oxidase synthase (iNOS) protein level expression analysis. Similarly, the second group of animals (*n* = 6, group LPS/AMS36) was treated with AMS36 dissolved in DMSO/saline solution ratio 1:10 (vol/vol) at a dose of 50 mg/kg (i.p.) 2 days prior to LPS/saline injection (into the right and left striatum, respectively) and then treated every fourth day for 4 weeks. After 4 weeks, the animals were sacrificed and brain tissue was further examined with histological staining and proteins were extracted for TH protein level expression analysis. Animals were killed with CO_2_. All experiments were performed during day time. A graphical outline of the experiment is presented in Supplementary Figure 3.

### Brain Tissue Preparation

Subjects of the group LPS (*n* = 5) and the group LPS/AMS36 (*n* = 6) were sacrificed after 4 weeks of LPS injection and brains were rapidly removed and quickly frozen on dry ice and stored at −80°C until sectioning on a cryostat. Of each subject, coronal brain sections (10–20 μm), identified using a rat brain atlas (Paxinos and Watson, 2007), were cut at four anterior-posterior levels through the striatum (in mm from bregma); (1) between 1.92 and 1.20, (2) between 1.08 and 0.24, (3) between 0.12 and −0.48, and (4) between −0.60 and −1.44 and at three anterior-posterior levels through the SNc (in mm from bregma); (1) between 4.68 and −5.04, (2) between −5.20 and −5.52, and (3) between −5.64 and −6.00. The sections were mounted onto microscope glass slides coated with a 0.01% solution of (poly)l-lysine (Sigma-Aldrich) or adhered to parafilm. The slides were then vacuum-packed and stored in a freezer at −20°C until being further processed.

### Methylene Staining

Slices (10 μm) were fixed in 4% formaldehyde in 0.1 M sodium phosphate buffer saline (NaPBS; pH 7.2–7.4, 4°C) for 5 min. After washing with potassium phosphate buffer saline (KPBS, pH 7.2), sections were counterstained with 0.2% methylene blue (Sigma-Aldrich) for 1 min, rinsed with distilled water, dehydrated in rising ethanol concentration and coverslipped with the DPX mounting medium (BDH Laboratory Supplies, Poole, UK).

### Lateral Ventricle and Striatum Area Quantification

Methylene blue-stained brain slices were scanned by MCID, M4 analyzer (Imaging Research, St Catharines, ON, Canada). Areas of lateral ventricles and striata were manually outlined and measured in striatal brain slices cut at 4 different anterior-posterior levels of LPS (*n* = 5) or LPS/AMS36 (*n* = 6) rat brains. The scan area (pixels) of lateral ventricle/striatum was separately measured in the contralateral and ipsilateral side. In the graphs sum of scan area of lateral ventricles/striata in striatal brain slices are shown.

### Immunohistochemistry

Coronal brain sections (10 μm) of LPS (*n* = 5) or LPS/AMS36 (*n* = 6) rat brains were fixed in cold 100% methanol for 5 min followed by 15 min in cold methanol with 1% H_2_O_2_. Brain slices were incubated in blocking buffer containing 4% normal serum, 1% bovine serum albumin (BSA), and 0.1% Triton X-100 in KPBS for 1 h at room temperature. They were then incubated overnight at 4°C with mouse monoclonal antibody against TH (1:750, Abcam, Cambridge, UK) or goat polyclonal primary antibody against cathepsin X (1:200, AF934, R&D Systems, MN, United States), diluted in blocking solution. Afterwards, the sections were incubated with biotinylated anti-mouse or anti-goat secondary antibodies (1:750, Vector Laboratories, Burlingame, CA, United States) diluted in KPBS containing 1% BSA and 0.02% Triton X-100 for 1.5 h at room temperature. Avidin-biotin-peroxidase complex (ABC elite standard kit, Vector Laboratories, Burlingame, CA, United States) was added for 30 min. Staining was visualized with 3,3′-diamino-benzidine (DAB, Sigma-Aldrich). All sections were simultaneously immunolabeled to ensure identical DAB staining incubation times. Sections were then dehydrated and coverslipped with DPX mounting medium (BDH Laboratory Supplies). Brain sections were scanned by MCID, M4 analyzer and examined and imaged with an Olympus microscope (Olympus IX81) with an attached digital camera (Olympus DP71) using the same system settings for all samples.

### *In situ* Hybridization

The standard procedure described by Zivin et al. was used for *in situ* hybridization histochemistry (Zivin et al., 1996). The 10 μm brain sections of LPS rats (*n* = 5) were incubated with 35S-labeled oligodeoxyribonucleotide “antisense” probes (45 bases long) complementary to the rat tyrosine-hydroxylase (TH) mRNA (sequence 5′-AAC CAA ACC AGG GCA CAC AGG GAG AAC CAT GCT CTT AAG-3′) and rat cathepsin X mRNA (sequence 5′-AGG TCT CAT CGG GGA TGC CAT GCT TGT GGG CAT ACT CCC ACA CCG-3′). The GenBank accession numbers used to design the probes were TH M23598 and CTSX NM183330. Hybridized sections were exposed to X-ray film (Scientific Imaging Film X-OmatTM AR, Kodak, Rochester, NY, United States) for 2–3 weeks and developed using standard darkroom techniques.

### Protein Extraction From Striatal and Nigral Sections

For analysis of the protein level of TH or cathepsin X and its activity, the striatum and SNc were dissected out from four 20 μm frozen brain slices (striatum) or eight 20 μm frozen brain slices (SNc) of LPS (*n* = 5) or LPS/AMS36 (*n* = 6) rat brains, separately from the contralateral (Control) and ipsilateral side (Lesion), using a cryostat at −20°C. Tissue was homogenized in ice-cold lysis buffer (0.05 M sodium acetate, pH 5.5, 1 mM EDTA, 0.1 M NaCl, 0.25% Triton X-100) supplemented with a cocktail of phosphatase inhibitors (Thermo Fisher Scientific, Waltham, MA, United States), then sonicated and centrifuged at 15.000 g at 4°C for 15 min to collect the supernatant. Total protein concentration was determined by DC™ Protein Assay (Bio-Rad, Hercules, CA, United States). All the samples were kept at −70°C until they were used for analysis.

### ELISA

The protein levels of cathepsin X in the striatum and SNc of LPS rat brains (*n* = 5) were determined by ELISA as previously reported (Kos et al., 2005). Briefly, microtiter plates were coated with equal aliquots of goat polyclonal anti-cathepsin X antibody (RD Systems) in 0.01 M carbonate/bicarbonate buffer, pH 9.6, at 4°C. After blocking with 2% BSA in phosphate-buffered saline (PBS), pH 7.4, for 1 h at room temperature, the samples of equal protein amount (50 μg) or cathepsin X standards (0–65 ng/mL) were added. Following 2 h incubation at 37°C, the wells were washed and filled with mouse monoclonal anti-cathepsin X 3B10 antibody conjugated with HRP in blocking buffer. Mouse monoclonal 3B10 antibodies were prepared from mouse hybridoma cell line as reported (Kos et al., 2005). After a further 2 h incubation at 37°C, 200 μL/well of 3,3′,5,5′-tetramethylbenzidine (TMB) substrate (Sigma-Aldrich) in 0.012% H_2_O_2_ was added. After 15 min, the reaction was stopped by adding 50 μL/well of 2 μM H_2_SO_4_. The amount of protein was determined by measuring the absorbance at 450 nm using a microplate reader (Tecan Safire^2^, Switzerland), and the concentration of cathepsin X was calculated from the standard calibration curve. Cathepsin X protein level was expressed relative to the contralateral striatum/SNc (Control).

### Cathepsin X Activity

Cathepsin X activity in the striatum and SNc of LPS rat brains (*n* = 5) was measured in tissue lysates with the cathepsin X-specific, intramolecularly quenched fluorogenic substrate Abz-Phe-Glu-Lys(Dnp)-OH synthesized by Jiangsu Vcare Pharmatech Co. (China). An aliquot of 50 μg of the lysate proteins was incubated at 37°C, followed by measurement of cathepsin X activity using 10 μM Abz-Phe-Glu-Lys(Dnp)-OH. The fluorometric reaction was quantified at 37°C at an excitation wavelength of 320 nm and emission wavelength of 420 nm on a microplate reader (Tecan Safire^2^). Results are presented as change in fluorescence as a function of time (ΔF/Δt) and cathepsin X activity was expressed relative to Control.

### Double Immunofluorescence Labeling

The striatal and nigral sections (10 μm) of LPS treated rats (*n* = 5) were double-immunostained for the analysis of the cellular localization of cathepsin X using the following primary antibodies: goat polyclonal anti-cathepsin X antibody (1:75, R&D System), mouse monoclonal anti-TH antibody (1:500, Abcam) as a marker for dopaminergic neurons, mouse monoclonal anti-NeuN antibody (1:300, EMD Millipore) as a neuronal marker, mouse monoclonal anti-CD11b antibody (1: 175, Abcam) as microglia marker and mouse monoclonal anti-glial fibrillary acidic protein (GFAP) antibody (1:1000, Abcam) as astrocyte marker. Brain slices were fixed in cold methanol for 20 min and the immunofluorescence procedure was further performed as reported (Tratnjek et al., 2016). Briefly, brain slices were incubated in blocking solution containing 4% donkey serum (EMD Millipore) and 0.4% Triton X-100 in KPBS for 1 h at room temperature. Brain sections were then incubated with the primary antibodies overnight at 4°C diluted in KPBS containing 1% donkey serum and 0.4% Triton X-100, followed by 1.5 h incubation at room temperature. Thereafter, sections were incubated with the Alexa-fluorophore-conjugated secondary antibodies (1: 300, Invitrogen, Molecular Probes, OR, United States) diluted in KPBS containing 0.02% Triton X-100 for 1.5 h at room temperature. After the incubation, sections were immersed into 0.1% Sudan Black B (Sigma-Aldrich) in 70% ethanol (vol/vol) for 5 min to suppress lipofuscein autofluorescence background. Sections were then rinsed with KPBS and coverslipped using the ProLong® Gold Antifade Mountant with DAPI (Thermo Fisher Scientific). To confirm staining specificity, primary antibodies were omitted in the staining procedure (*data not shown*). Fluorescence microscopy was performed using a Carl Zeiss LSM 710 confocal microscope (Carl Zeiss, Oberkochen, Germany). Images are presented as single confocal sections and were analyzed using Carl Zeiss ZEN 2011 image software. All images were captured under the same exposure time settings. To improve the signal/noise ratio, 4 frames/image were averaged.

### Western Blotting

The protein level of TH and iNOS in the striatum of LPS (*n* = 5) and LPS/AMS36 (*n* = 6) rat brains was determined by western blotting as previously reported (Hafner et al., 2012). Briefly, equal aliquots of the protein (30 μg per lane) were resolved on 12% SDS-PAGE. Separated proteins, transferred to a nitrocellulose membrane were incubated with 5% non-fat dried milk in TBST buffer (Tris-buffered saline with 0.1% Tween 20; 20 mM Tris, 137 mM NaCl, pH 7.4, containing 0.1% Tween-20) at room temperature for 1 h to block non-specific binding. Designated proteins were immunoblotted with the rabbit monoclonal anti-TH antibody (1:400, Abcam), rabbit polyclonal anti-iNOS (1:500, Abcam), and mouse monoclonal anti-β-actin antibody (1:500, Sigma Aldrich) in TBST containing 3% BSA, incubated overnight at 4°C. The membranes were washed three times for 10 min in TBST, incubated for 1 h with horseradish peroxidase (HRP)-conjugated secondary antibodies and finally washed six times for 10 min with TBST. Signals from anti-rabbit conjugated with HRP or anti-mouse conjugated with HRP (1:5000, Millipore, Billerica, MA, United States) secondary antibodies were visualized with an enhanced chemiluminescence detection kit (Thermo Scientific, Rockford, IL, United States). The band intensities were quantified using Gene Tools software (Sygene, UK), and expressed as values relative to those of controls.

### Statistical Analysis

Statistical evaluation was performed with the two-tailed Student's *t*-test when two values sets were compared, where *p* < 0.05 was considered to be statistically significant. The differences in striatum area or size of lateral ventricles between contralateral and ipsilateral sides within each experimental group [LPS (*n* = 5), LPS/AMS36 (*n* = 6)] were analyzed using ANOVA test and *post hoc* analysis using Student's *t*-test. When two sets of values were compared; *p* < 0.05 was considered to be statistically significant. For all the statistical evaluation, GraphPad Prism (version 6) was used.

## Results

### Neuroinflammation-Induced Cathepsin X Expression in the Brain

**Figure 1:**
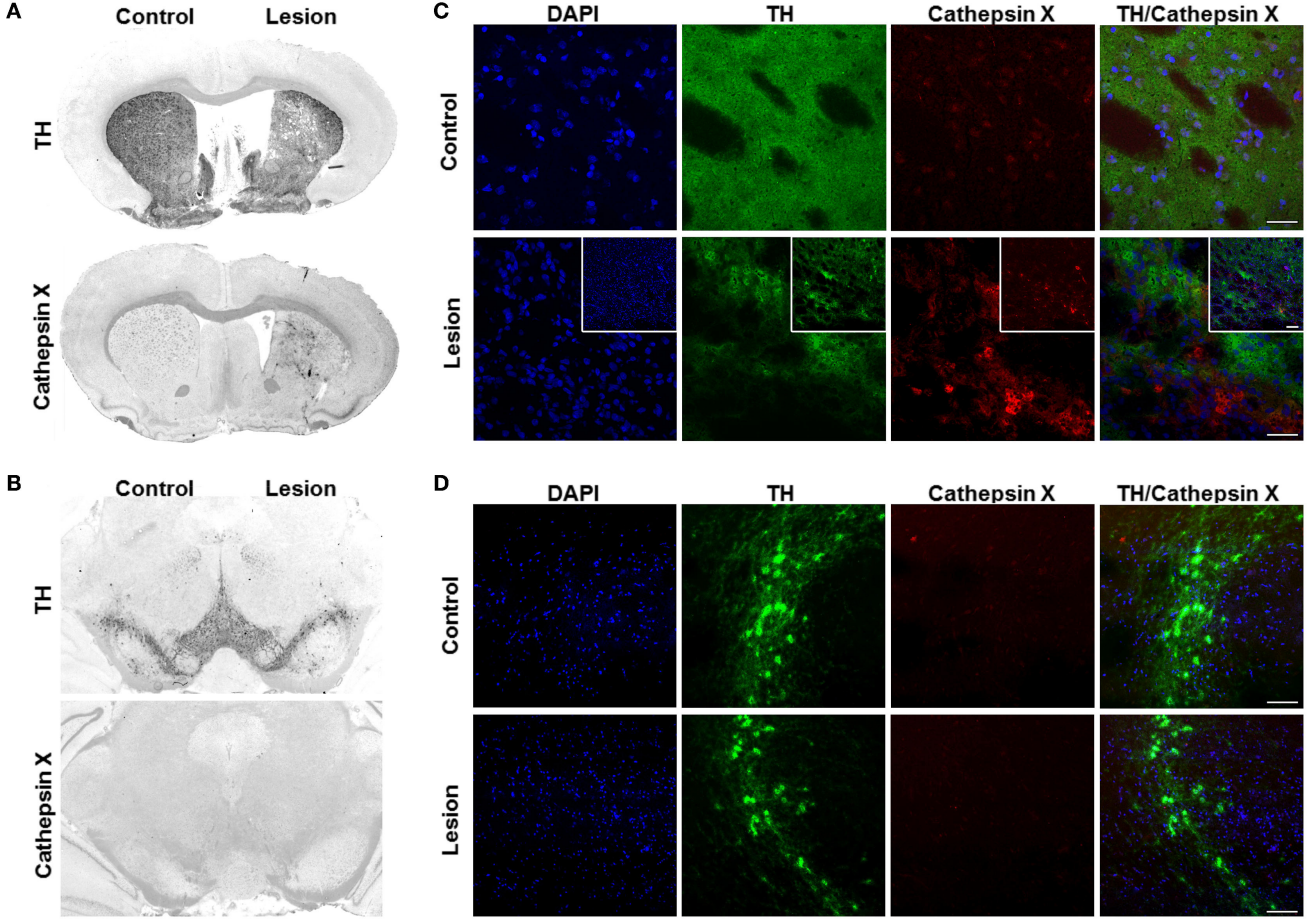
Cathepsin X upregulation in the striatum after intrastriatal LPS injection. **(A,B)** Representative immunohistochemical images of coronal sections of the striatum **(A)** and SNc **(B)** from the 4 weeks' time-point after LPS-induced lesion. TH staining demonstrated decrease of TH-immunostaining in the striatum **(A)** and no loss of TH-positive cells in the SNc (**B**, *upper panel*) in the ipsilateral side (Lesion) compared to contralateral side (Control), whereas cathepsin X immunoreactivity was markedly increased in the ipsilateral striatum after LPS injection **(A)**. No increase in cathepsin X immunoreactivity was observed in the ipsilateral SNc after LPS-induced lesion (**B**, *lower panel*). **(C,D)** Representative images of double immunofluorescence staining of TH (green fluorescence) and cathepsin X (red fluorescence) in the striatum **(C)** and SNc **(D)** at 4 weeks after the LPS injection. Nuclei were counterstained with DAPI (blue fluorescence). In the ipsilateral (Lesion) striatum, TH-immunoreactivity was reduced and TH-staining is patchy compared to the contralateral side (Control), whereas cathepsin X expression was upregulated in the ipsilateral side of striatum after LPS injection **(C)**. The insets show a double immunofluorescence staining of TH (green fluorescence) and cathepsin X (red fluorescence) in the lesioned striatum at lower magnification **(C)**. In the SNc, no upregulation of cathepsin X in the ipsilateral side was observed **(D)**. Group of 5 animals was conducted (*n* = 5, group LPS), where 4 anterior-posterior striatal slices and 3 nigral anterior-posterior slices of each animal were analyzed. *Scale bars* = *20* μm **(C,D)**, *100* μm (insets, **C**).

Rats were unilaterally lesioned by injection of LPS into the right striatum to evaluate cathepsin X expression pattern in the brain with the inflammation-induced neurodegenerative pathology. Vehicle was injected in parallel coordinates into the left striatum. There was a reduction in striatal size and increase in lateral ventricle size in the ipsilateral hemisphere following LPS injection (Figures 1A, **7A–C**). Immunohistochemical analysis of TH expression, a dopaminergic neuronal marker, revealed decreased TH-immunoreactivity in the ipsilateral striatum after LPS injection compared to the contralateral side (Figure 1A), while there were no changes in TH immunostaining intenstiy between the ipsilateral and contralateral SNc (Figure 1B). No significant loss of dopaminergic neurons in the SNc in the ipsilateral hemisphere was confirmed with *in situ* hybridization method (Supplementary Figure 4).

The results of immunohistochemical analysis of cathepsin X showed an increase of protein expression in the ipsilateral striatum and surrounding areas (Figure 1A), whereas no increase in cathepsin X immunoreactivity was observed in the ipsilateral SNc 4 weeks after LPS injection (Figure 1B). Similarly, cathepsin X mRNA level was markedly increased in the ipsilateral striatum after LPS injection (Supplementary Figure 4). Vehicle injection in the contralateral striatum on the other hand did not increase cathepsin X protein or mRNA levels (Figure 1A, Supplementary Figure 4). Furthermore, immunofluorescence staining of the ipsilateral striatum showed decreased and patchy TH-immunoreactivity, with a concomitant marked increase of cathepsin X expression (Figure 1C), and no upregulation of cathepsin X expression in the ipsilateral SNc, which coincides with no change in TH-immunostaining intensity in the ipsilateral SNc when compared to the contralateral side (Figure 1D).

**Figure 2:**
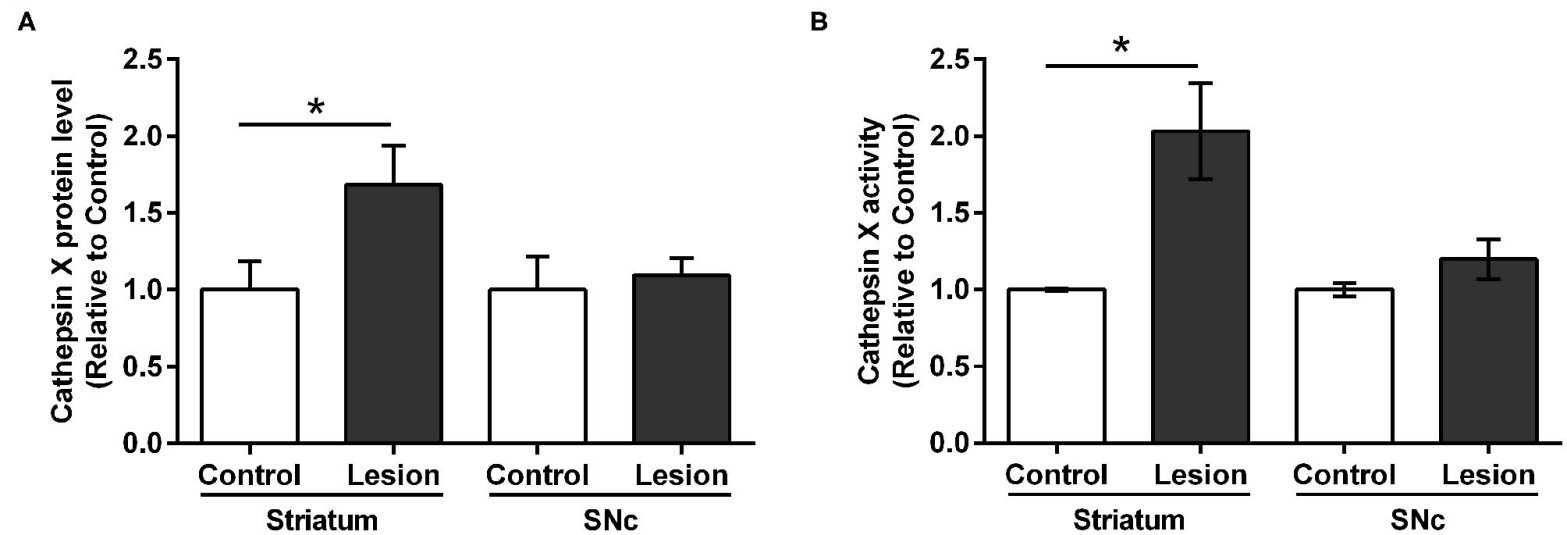
Cathepsin X protein level and activity in the striatum and SNc following intrastriatal LPS injection. Four weeks after the intrastriatal induced LPS lesion, the contralateral (Control) and ipsilateral (Lesion) striatum and SNc were dissected from coronal sections, homogenized, and analyzed for cathepsin X protein levels and activity. **(A)** ELISA analysis of the cathepsin X protein level in the striatum and SNc after LPS-induced lesion. Cathepsin X protein level in the ipsilateral striatum was significantly increased compared to the contralateral side, whereas no significant difference in protein level in the SNc was observed. **(B)** Enzymatic activity analysis shows an increase in cathepsin X activity in the lesioned striatum after LPS injection, whereas the increase in cathepsin X activity in the ipsilateral SNc was not significant compared to the activity in contralateral SNc. Values are means ± SD of group of 5 animals (*n* = 5, group LPS), where 4 anterior-posterior striatal slices and 8 nigral anterior-posterior slices of each animal were analyzed (two-tailed Student's *t-*test, *p<0.05 vs. Control).

Furthermore, we additionally characterized the used hemi-LPS model by analysis of the TH and iNOS protein level expression. iNOS is expressed in microglia during neuroinflammation and produces an excessive amount of nitric oxide, which can cause the death of dopaminergic neurons (Bal-Price and Brown, 2001). The expression patterns of TH and iNOS were examined in the contralateral and ipsilateral striatum following intrastriatal LPS injection by western blot analysis. LPS injection caused a significant decrease in TH protein level in the ipsilateral striatum compared to contralateral, whereas increased striatal iNOS was observed at the ipsilateral striatum compared to contralateral (Supplementary Figure 5). Based on the immunostaining observations of cathepsin X upregulation after LPS-induced lesion, proteins were isolated from the dissected contralateral and ipsilateral striatal and SNc regions of coronal sections in order to confirm changes in cathepsin X protein level and its enzymatic activity using quantitative methods. ELISA analysis revealed a prominent increased expression of cathepsin X protein level in the ipsilateral striatum compared to the contralateral striatum; however, no significant difference in the expression of cathepsin X was seen between the ipsilateral and contralateral SNc (Figure 2A). Furthermore, analysis of protein activity showed that the LPS-induced lesion also significantly increased the enzymatic activity of cathepsin X in the ipsilateral striatum relative to contralateral (Figure 2B). Similarly, no significant increase in cathepsin X activity was detectable in the ipsilateral SNc compared with the enzyme activity in the contralateral SNc (Figure 2B), which is consistent with the results of cathepsin X protein level in the SNc obtained by ELISA analysis. Taken together, these observations indicate that the LPS-induced striatal degeneration is accompanied by an increase in cathepsin X expression and its activity.

### Distribution of Brain Cathepsin X Following LPS Injection

**Figure 3:**
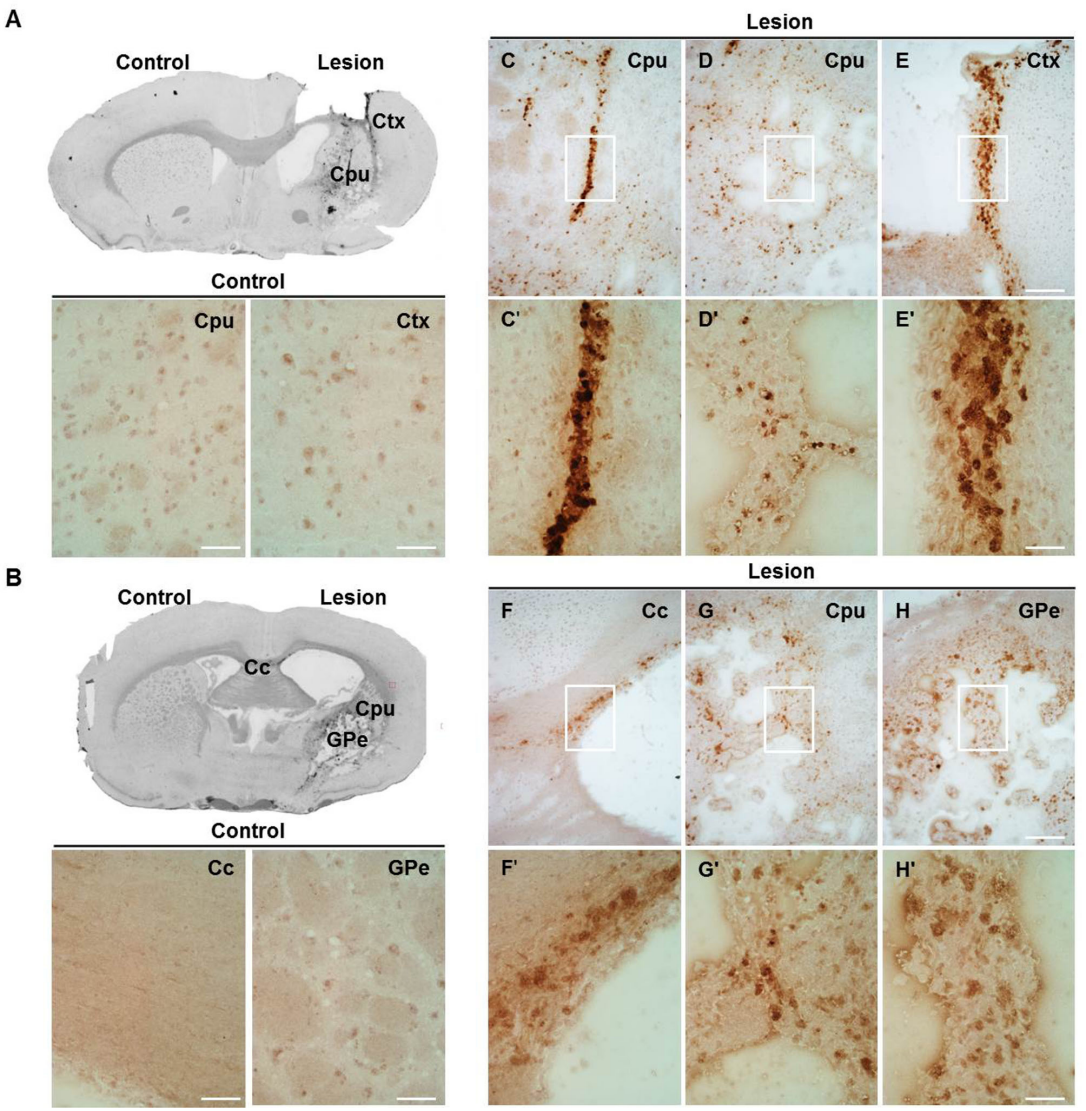
The expression pattern of upregulated cathepsin X after LPS injection. Representative cathepsin X immunohistochemical images of the contralateral (Control) and ipsilateral (Lesion) sides of the striatal brain slices 4 weeks after LPS-induced lesion. **(A,B)** On the contralateral side, weak signal for endogenous cathepsin X in the striatum (caudate-putamen, Cpu) or surrounding areas was observed. **(C–H)** Cathepsin X staining demonstrated that cathepsin X expression was strongly induced in the ipsilateral striatum (Cpu; **C,D,G**) and throughout the different areas, such as the ipsilateral cortex (Ctx; **E**), corpus callosum (Cc; **F**), and external globus pallidus (GPe; **H**). The white box areas in images **C–H** are shown in magnified view in images **C′–H′**. Group of 5 animals (*n* = 5, group LPS) was conducted, where 4 anterior-posterior striatal slices of each animal were analyzed. *Scale bar* = 200 μm **(C–H)**; 50 μm **(A,B,C′–H′)**.

In order to more precisely define the distribution of upregulated cathepsin X in LPS-induced neuroinflammation, we performed additional immunohistochemical staining of cathepsin X on the striatal brain cryosections. Weak cathepsin X staining was detected in the contralateral striatum and throughout different areas such as the cerebral cortex (Ctx), corpus callosum (Cc), and external globus pallidus (GPe) (Figures 3A,B). However, an abundant increase in cathepsin X expression was observed predominantly in the ipsilateral striatum (Figures 3C–G), where condensed expression of cathepsin X was apparent (Figure 3C′) or scattered throughout the striatum region (Figures 3D′,3G′). Strong upregulation of cathepsin X expression was also observed in the ipsilateral Ctx (Figure 3E) and Cc (Figure 3F) around the injection track, where dense cathepsin X staining was observed. Nevertheless, a marked increase in cathepsin X expression was also observed in the ipsilateral GPe (Figure 3H) with scattered distribution through the region. Thus, the results of altered cathepsin X expression and distribution following LPS injection indicate relevant biological function of the protein in the brain under pathological conditions.

### Cellular Localization of Brain Cathepsin X Following LPS Injection

**Figure 4:**
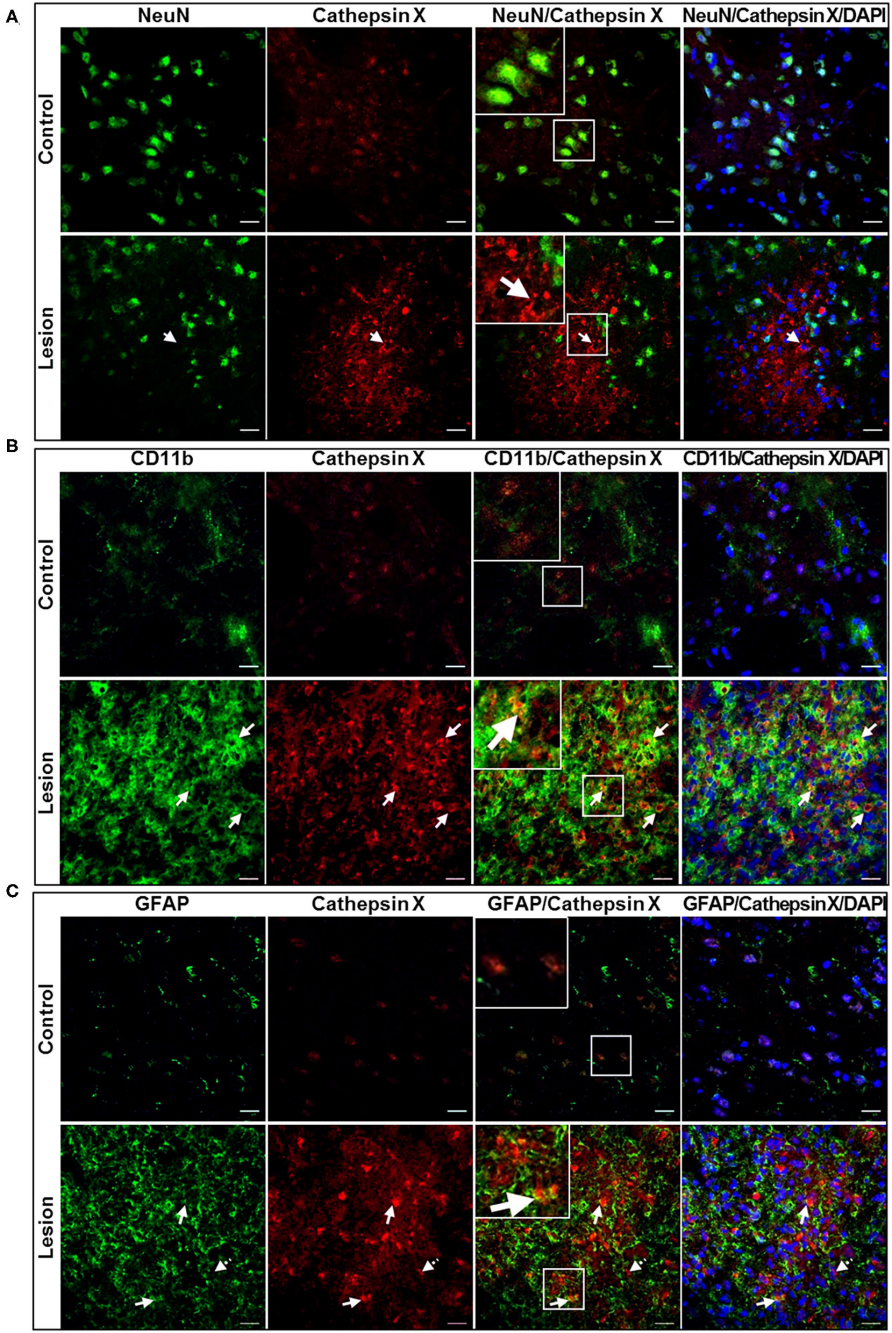
Cell-specific localization of cathepsin X in the striatum with LPS-induced lesion. **(A–C)** Representative images of double immunofluorescent staining of cathepsin X (red fluorescence) and cell-type markers (green fluorescence) for neurons (NeuN, **A**), microglial cells (Cd11b, **B**) and astrocytes (GFAP, **C**) in the contralateral (*upper panel*) and ipsilateral (*lower panel*) striatum at 4 weeks after the LPS injection. Nuclei were counterstained with DAPI (blue fluorescence). In the lesioned striatum, cathepsin X was predominantly restricted to CD11b-positive cells (**B**, *arrows in lower panel*) and GFAP-positive cells (**C**, *arrows in lower panel*), whereas neuronal cells were not positive for upregulated cathepsin X (**A**, *arrow in lower panel*). The opposite, the endogenously expressed cathepsin X is present in NeuN-positive cells (**A**, *upper panel*). Group of 5 animals (*n* = 5, group LPS) was conducted, where 4 anterior-posterior striatal slices of each animal were analyzed. *Scale bar* = 20 μm.

**Figure 5:**
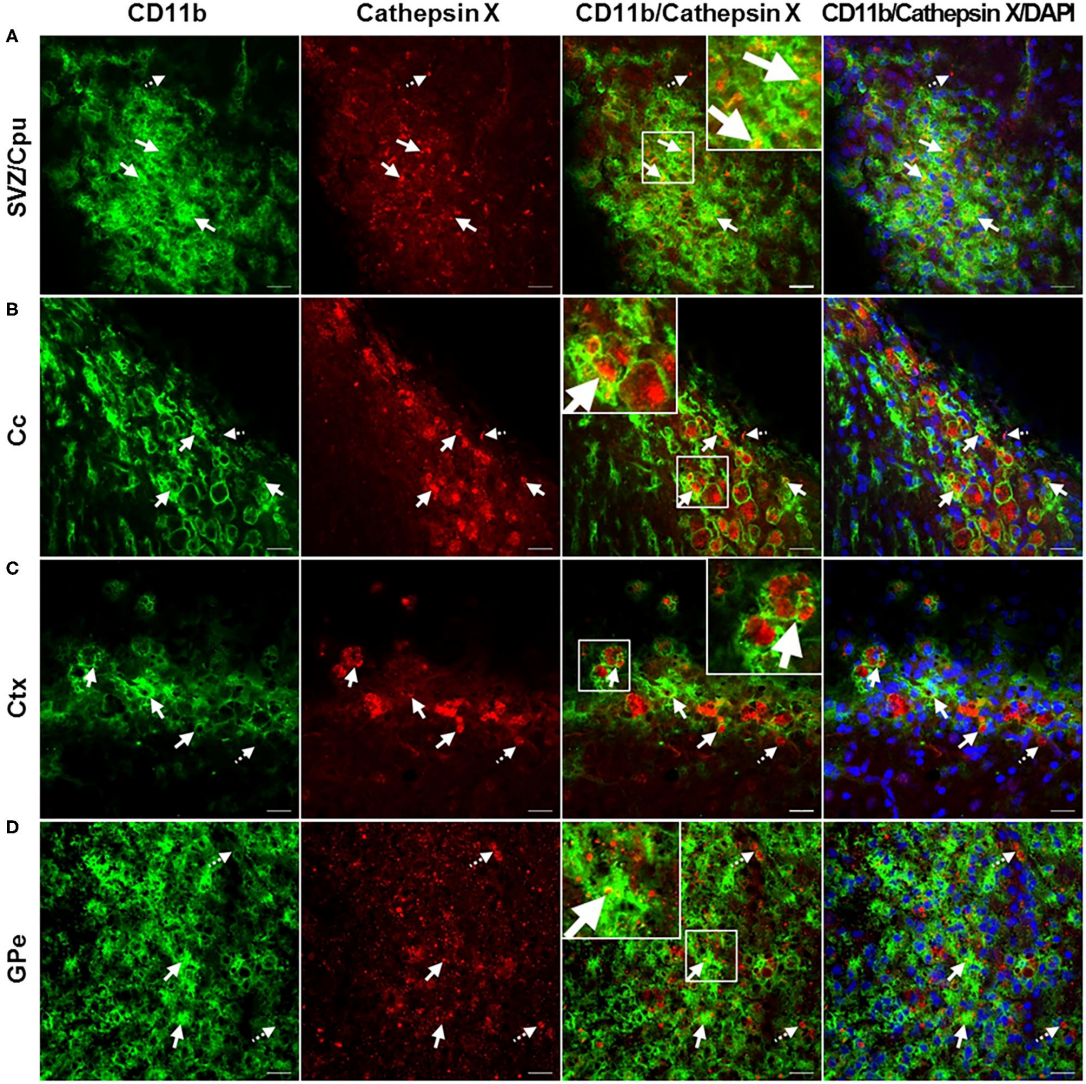
Microglial phenotype of increased cathepsin X-immunopositive cells after intrastriatal LPS injection. **(A–D)** Representative images of double immunofluorescence staining of microglial marker CD11b (green fluorescence) and cathepsin X (red fluorescence) in the ipsilateral side of the striatal brain slices 4 weeks after LPS-induced lesion. Nuclei were counterstained with DAPI (blue fluorescence). In the subventriclular zone/caudate-putamen (SVZ/Cpu; **A**), corpus callosum (Cc; **B**), cortex (Ctx; **C**), and external globus pallidus (GPe; **D**) upregulated cathepsin X was predominantly restricted to CD11b-positive cells (arrows), however, some cathepsin X-positive signal did not overlap with CD11b-immunosignal in SVZ/Cpu and GPe (dashed arrows). Group of 5 animals (*n* = 5, group LPS) was conducted, where 4 anterior-posterior striatal slices of each animal were analyzed. Scale bar = 20 μm.

**Figure 6:**
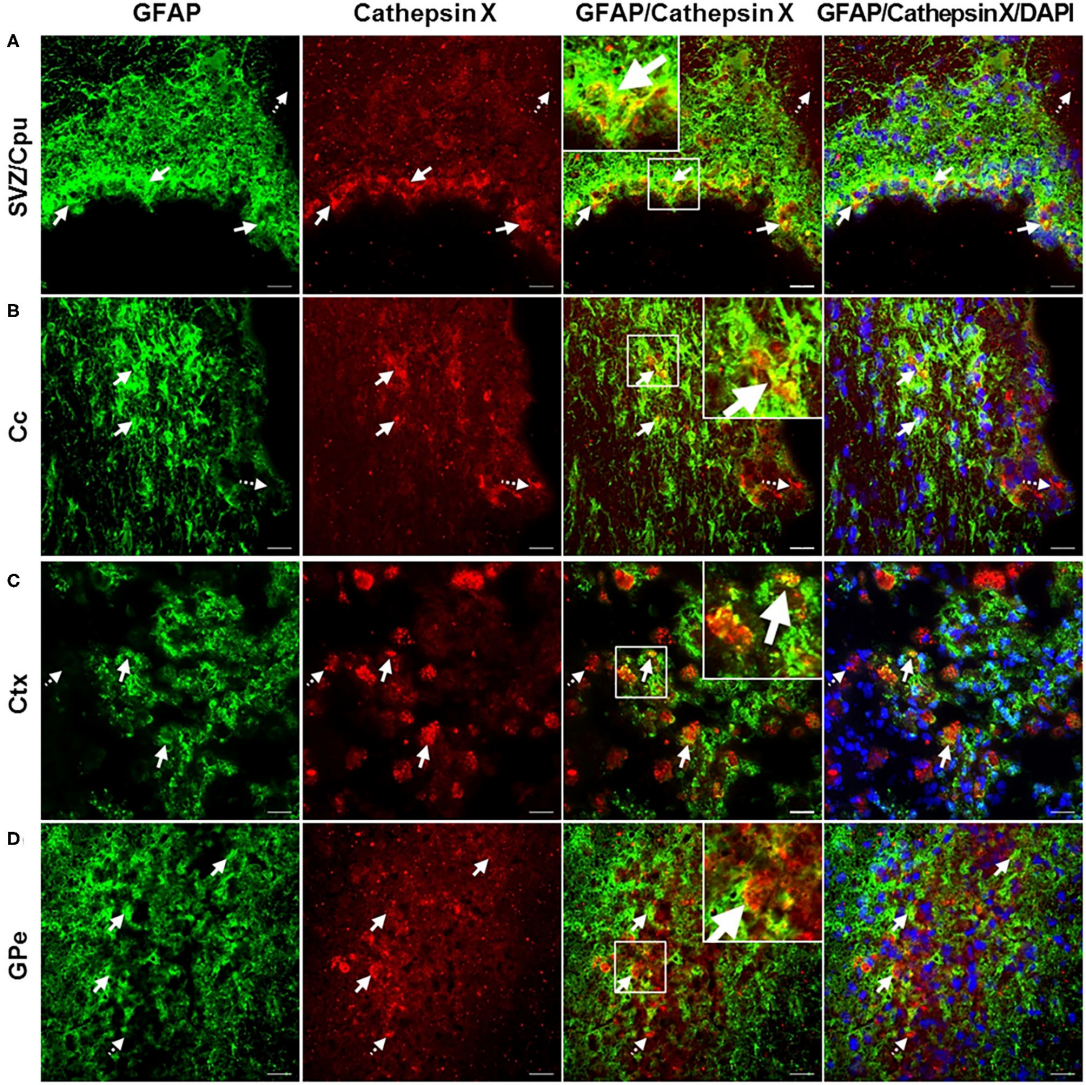
Astroglial phenotype of increased cathepsin X-immunopositive cells after intrastriatal LPS injection. **(A–D)** Representative images of double immunofluorescence staining of marker for astrocytes GFAP (green fluorescence) and cathepsin X (red fluorescence) in the ipsilateral side of striatal brain slices 4 weeks after LPS-induced lesion. Nuclei were counterstained with DAPI (blue fluorescence). In the subventriclular zone/caudate-putamen (SVZ/Cpu; **A**), corpus callosum (Cc; **B**), cortex (Ctx; **C**), and external globus pallidus (GPe; **D**) increased cathepsin X expression was partially observed in GFAP-positive cells, predominantly in SVZ/Cpu area (arrows); however in all regions cathepsin-positive signal was also seen in GFAP-negative cells (dashed arrows). Group of 5 animals (*n* = 5, group LPS) was conducted, where 4 anterior-posterior striatal slices of each animal were analyzed. Scale bar = 20 μ.

In order to further explore which type of cells express cathepsin X following LPS injection, we conducted double immunofluorescent staining to detect the co-localization of cathepsin X with different cell-specific markers, including markers for neurons (NeuN), microglial cells (CD11b) and astrocytes (GFAP). LPS injection induced activation of microglia and astrocytes, as indicated by an increased number of rounded, amoeboid microglia and astrocytes in the ipsilateral striatum (Figures 4B,C). Microgliosis in the LPS-lesioned striatum demonstrated with double immunolabeling is in accordance with increased iNOS levels obtained with western blotting (Supplementary Figure 5). Endogenously expressed cathepsin X in the contralateral striatum was mainly present in NeuN-positive cells (Figure 4A, *upper panel*). However, LPS-induced cathepsin X expression was not upregulated in NeuN-positive cells in the ipsilateral striatum (Figure 4A, *lower panel*) or in other areas such as the subventricular zone (SVZ), Ctx, Cc and GPe (see Supplementary Figure 6). Upregulated cathepsin was restricted to glial cells. Cathepsin X staining was localized mainly within microglia cells in the ipsilateral striatum (Figure 4B, *lower panel*) and throughout different areas including the SVZ (Figure 5A), Cc (Figure 5B), Ctx (Figure 5C) and GPe (Figure 5D) as demonstrated by co-localization of cathepsin X with CD11b. In the striatum, strong co-localization of cathepsin X with GFAP was also observed at the ipsilateral side (Figure 4C, *lower panel*) and in the SVZ at the ipsilateral side (Figure 6A). Additionally, as shown in Figure 6 some astrocytes in the ipsilateral Cc (Figure 6B), Ctx (Figure 6C) and GPe (Figure 6D) also showed cathepsin X overexpression. Nevertheless, after 4 weeks of LPS-induced lesion, no localization of physiologically expressed cathepsin X was observed in glial cells in the contralateral striatum probably due to minimal glial reactivity, where only individual CD11b- and GFAP-positive cells were observed (Figures 4B,C, *upper panels*). These results further suggest the significant role of cathepsin X in neurodegeneration that is associated with glial activation.

### Evaluation of *in vivo* Potency of Cathepsin X Inhibitor

**Figure 7:**
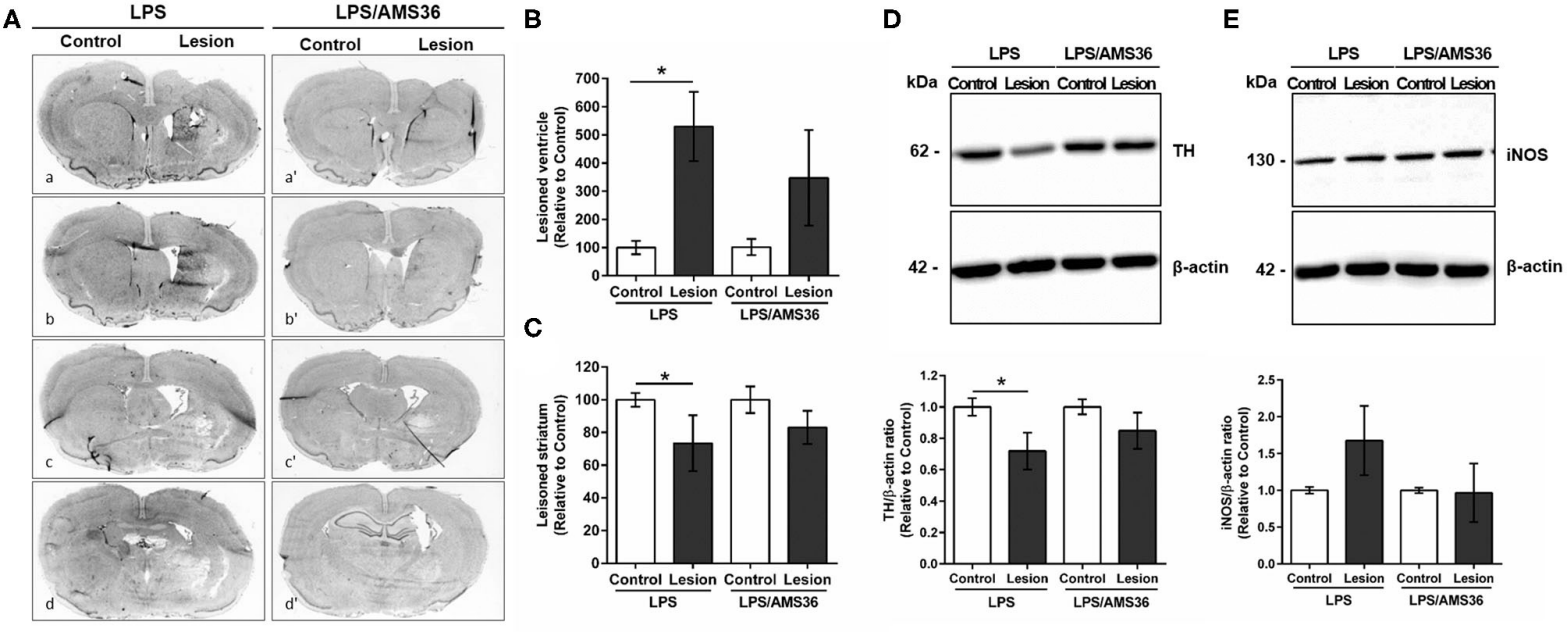
Cathepsin X inhibitor alleviated degeneration following intrastriatal LPS injection. **(A)** Representative images of methylene blue staining of coronal sections of the striatum cut at four anterior-posterior levels; between 1.68 and 1.28 mm from bregma **(a,a′)**; between 0.96 and 0.56 mm from bregma **(b,b′)**; between 0.00 and −0.40 mm from bregma **(c,c′)**; between −0.40 and −1.40 mm from bregma **(d,d′**), 4 weeks after LPS induced lesion in the absence of cathepsin X inhibitor AMS36 (LPS) or the presence of AMS36 (LPS/AMS36). **(B)** The graph shows the analysis of the size of the lateral ventricle as a sum of lateral ventricle areas in 4 anterior-posterior striatal slices at the ipsilateral side (Lesion) relative to contralateral side (Control). Values are means ± SD of group of 5 animals for LPS (*n* = 5) and group of 6 animals for LPS/AMS36 (*n* = 6), where 4 anterior-posterior striatal slices from each animal were analyzed (*n* = 5/6) (ANOVA, Student's *t*-test, *p<0.5 Control vs. Lesion). **(C)** The graph shows the analysis of the size of striatum as a sum of striatum areas in 4 anterior-posterior striatal slices at the ipsilateral side relative to contralateral side. Values are means ± SD of group of 5 animals for LPS (*n* = 5) and group of 6 animals for LPS/AMS36 (*n* = 6), where 4 anterior-posterior striatal slices from each animal were analyzed (*n* = 5/6) (Student's *t*-test, *p<0.5 Control vs. Lesion). **(D,E)** Western blot analysis of TH and iNOS expression in the contralateral (Control) and ipsilateral (Lesion) dissected striatal sections after LPS-induced lesion in the absence of cathepsin X inhibitor AMS36 (LPS, *n* = 5) or the presence of AMS36 (LPS/AMS36, *n* = 6), using rabbit monoclonal anti-TH antibody **(D)** or rabbit polyclonal anti-iNOS antibody **(E)**, respectively. An antibody raised against β-actin was used as loading control. The graphs below blot shows a semi-quantitative densitometry analysis of the protein level in the ipsilateral side relative to that in the contralateral side. Values are means ± SD of group of 5 animals for LPS (*n* = 5) and group of 6 animals for LPS/AMS36 (*n* = 6), where 4 anterior-posterior striatal slices of each animal were analyzed (*n* = 5/6) (two-tailed Student's *t*-test, *p<0.05 vs. Control).

Intrigued by the results of strongly increased cathepsin X expression and its activity after LPS-induced lesion, we were interested whether the inhibition of cathepsin X enzymatic activity might be useful in preventing the striatal degeneration. We performed a preliminary analysis to determine the potency of cathepsin X inhibition by specific inhibitor AMS36 *in vivo*. After the inhibitor administration, decreased cathepsin X activity was observed in cerebellum extracts; however, a significant decrease in cathepsin X activity was detectable after 2 days of administration (see Supplementary Figure 2). Therefore, to determine the potential effect of AMS36 on LPS-induced striatal degeneration, AMS36 was administrated 2 days prior to LPS injection and then every 4 days within 4 weeks. In LPS injected rats lateral ventricles were significantly dilated in the ipsilateral side compared to the contralateral side. Concomitantly, striatum size was reduced in the ipsilateral side (Figures 7A–C). Nevertheless, AMS36 attenuated the LPS-mediated dilation of the lateral ventricles and reduction in striatum size (Figures 7A–C). There were no longer statistical differences in lateral ventricle size between the contralateral and ipisalateral brain hemisphere when AMS36 was administered following the LPS injections (Figures 7A,B). Additionally, there was a trend toward a decreased extent of striatal lesion when AMS36 was administrated following LPS injection compared to LPS injection alone (Figure 7C). Moreover, cathepsin X inhibitor also affected TH protein level after LPS-induced lesion. LPS injection caused a significant decrease in TH protein level in the ipsilateral striatum compared to contralateral, whereas the significant decrease in TH protein expression was no longer observed when AMS36 was administrated (Figure 7D).

Finally, as we observed strong co-localization of cathepsin X with activated microglia marker CD11b in the ipsilateral striatum and throughout other areas, we were prompted to determine the iNOS protein level in lesioned striatum in AMS36-treated rats. iNOS is expressed in microglia during neuroinflammation and produces excessive amount of nitric oxide, which can cause the death of dopaminergic neurons (Bal-Price and Brown, 2001). Therefore, the expression pattern of iNOS was examined in the ipsilateral striatum following intrastriatal LPS injection, in the presence or absence of inhibitor AMS36 after 4 weeks administration. The increased striatal iNOS protein level in the ipsilateral striatum following LPS injection compared to contralateral striatum was abolished when AMS36 was continuously administrated together with LPS injection (Figure 7E). Together these results indicate a potential protective effect of cathepsin X inhibitor against striatal degeneration, which also supports a significant role of glial cathepsin X in neuroinflammation-induced neurodegeneration.

## Discussion

In this study, we utilized a model of neuroinflammation-induced degeneration of the rat brain by intrastriatal injection of LPS, and aimed to evaluate the regional and cellular expression and activity of lysosome cysteine cathepsin X. We described for the first time that cathepsin X expression and its activity are strongly upregulated *in vivo*, i.e., in the rat brain striatum after LPS-induced lesion. Along with striatal upregulation, cathepsin X overexpression was also found in the cerebral cortex, corpus callosum, subventricular zone and external globus pallidus, and in all of these regions, the upregulation was restricted to glial cells. Moreover, we explored the biological role of cathepsin X in neuroinflammation by cathepsin X-specific inhibitor AMS36. The latter indicated moderate protective effect toward the striatal lesion, suggesting that cathepsin X might play a significant role in neuroinflammation related to neurodegeneration.

Over the past decade, increasing evidence has emerged that implicates cathepsin X in the neurodegeneration. High cathepsin X levels have been observed in degenerating brain regions of amyotrophic lateral sclerosis and AD transgenic mouse models and around senile plaques in brains of AD patients (Wendt et al., 2007; Hafner et al., 2013). Furthermore, a comprehensive comparative gene expression analysis of mouse models of AD, multiple sclerosis and stroke showed that cathepsin X is one of the eighteen genes whose expression is increased in all three models of CNS disorders (Tseveleki et al., 2010). Cathepsin X has also been associated with inflammation processes leading to neurodegeneration. It has been shown that cathepsin X is disproportionately expressed and secreted by microglia and astrocytes in response to neuronal damage and inflammatory stimulus, both in culture and *in vivo* (Glanzer et al., 2007; Wendt et al., 2009; Greco et al., 2010; Pislar et al., 2017). It was reported that dendritic cells in aging mice brains show increased expression of cathepsin X protein level that correlates with known markers of neuroinflammation (Stichel and Luebbert, 2007). Additionally, Allan et al. (2017) showed that mice deficient in cathepsin X have reduced neuroinflammation and dramatically decreased circulating levels of interleukin 1β during experimental autoimmune encephalomyelitis. We recently showed the upregulation of cathepsin X in degenerated rat brain using a 6-OHDA rat PD experimental model. Unilateral 6-OHDA-induced lesion of the nigrostriatal pathways rapidly increased cathepsin X expression and activity in the ipsilateral SNc, which was mainly localized in TH-positive dopaminergic neurons. The effect was observed at 12 h of 6-OHDA injection, whereas a late time point of 6-OHDA-induced lesion, namely 4 weeks, caused persistent cathepsin X upregulation restricted to activated glia cells (Pislar et al., 2018). Thus, cathepsin X could be involved in neuroinflammation-induced dopaminergic neurodegeneration.

In this study, we adopted the LPS induced neuroinflammation rat model that induces neuronal loss thorugh microglial activation (Choi et al., 2009). Unilateral injection of LPS into the striatum leads to neuroinflammation-induced progressive dopaminergic neurodegeneration, which is accompanied by a progressively decreased number of TH-positive SNc cells, as well as by decreased TH-positive fibers in the striatum and decreased striatal DA levels (Choi et al., 2009; Hunter et al., 2009). In our study, intrastriatal LPS injection induced striatal degeneration, which was observed by the reduction of striatum and increased size of the lateral ventricle 4 weeks after injection. In addition, a marked decrease of TH-immunostaining in the ipsilateral striatum was observed; however, there was no obvious loss of TH-positive cells in the ipsilateral SNc following intrastriatal LPS injection. The latter observation is consistent with the study of Hoban et al. (2013), who showed that intrastriatal LPS administration did not lead to any nigrostriatal neurodegeneration. However, rats with instrastriatal LPS injection rotated significantly after amphetamine administration, a behavioral indicator of induced functional alterations in the nigrostriatal dopaminergic system on the ipsilateral side. Thus, LPS treatment enhances responding to amphetamine after LPS treatment, suggesting that LPS administration dysregulates presynaptic DA levels, post-synaptic DA receptor sensitization or alterations in DA turnover (Fortier et al., 2004; Fan et al., 2011; Hoban et al., 2013).

Furthermore, it is important to stress out that the serotype, route of administration, and number of injections of LPS induce varied pathological responses (Batista et al., 2019). Animals can respond to LPS stimuli differently depending on age and species. In addition, the source of the stimulus, the dose, the route, and the duration of the administration used in each study may also influence the outcome (Reviewed in Zakaria et al., 2017). Nevertheless, there is a certain reproducibility between different laboratories and studies. In agreement with other studies (Hunter et al., 2007; Choi et al., 2009; Hoban et al., 2013) we have shown microgliosis, astrogliosis, decreased striatal TH levels and pro-inflammatory effector enzyme iNOS induction in the ipsilateral brain hemisphere of LPS injection, which together indicate the model suitability for neuroiflammation studies.

Whilst the expression and release of cathepsin X has been shown to be associated with neuroinflammation (Glanzer et al., 2007; Wendt et al., 2009; Greco et al., 2010; Pislar et al., 2017), this study provides the first experimental evidence that cathepsin X is strongly upregulated in the rat brain following LPS-induced striatal inflammation leading to neurodegeneration. We showed a marked increase in protein expression in the ipsilateral striatum and surrounding areas 4 weeks after LPS injection. Likewise, TH staining showed that there is probably no loss of dopaminergic cells in the ipsilateral SNc, as well as no change in cathepsin X immunoreactivity in the ipsilateral SNc. Additionally, we showed that cathepsin X protein level was significantly increased in the striatum on the lesioned side as compared to the contralateral striatum, where the pro-form of upregulated cathepsin X was most prominent. Moreover, our enzyme assays showed a significant increase of cathepsin X activity in the ipsilateral striatum following LPS injection, which suggests that cathepsin X might display a significant proteolytic activity in the analyzed brain region during neuroinflammation-induced neurodegeneration.

Alterations in the expression patterns and localization of the lysosomal cathepsins in the CNS have been reported in normal and aged brain, and under pathological conditions (reviewed in Pislar and Kos, 2014). Cathepsin X expression and proteolytic activity were detected in different brain regions, such as the cerebral cortex, the cerebellum, the brainstem and the spinal cord in the adult mouse brain, and were found to be age-dependently upregulated during degenerative processes throughout brain regions (Wendt et al., 2007). Here, we examined the abundance and the regional distribution of cathepsin X in the lesioned striatum following LPS-induced neuroinflammation. Our immunohistochemical data show an abundant increase in cathepsin X expression predominantly in the ipsilateral striatum where condensed expression of cathepsin X was apparent or scattered throughout the striatum region. Nevertheless, strong upregulation of cathepsin X was also observed throughout different areas, such as in the cerebral cortex and corpus callosum around the injection track and in the external globus pallidus, where scattered distribution of cathepsin X through the region was observed. Thus, our data add another enzyme to the group of cysteine cathepsins, containing cathepsin B (Yan et al., 2013), L (Xu et al., 2018), H (Fan et al., 2015) and C (Fan et al., 2012), that have been reported to be upregulated in different brain regions following LPS-induced neuroinflammation.

Several lines of evidence support the hypothesis that glial reactive and inflammatory processes contribute to the cascade of events leading to neuronal degeneration (reviewed in More et al., 2013). We have shown that LPS injection into the striatum causes striatal microgliosis, and the presence of reactive astrocytes and we found that the majority of cathepsin X-immunoreactive signals were localized in these glial cells in the area of the lesioned striatum. Cathepsin X staining was localized mainly within microglia cells in the striatum and throughout different areas including the cerebral, corpus callosum, subventricular zone and external globus pallidus. This observation correlates well with previous *in vitro* studies showing upregulated expression, increased release and activity of microglial cathepsin X following LPS stimulation (Wendt et al., 2009; Pislar et al., 2017). We also observed cathepsin X upregulation in reactive astrocytes, where stronger co-localization with astrocyte marker was observed in the subventricular zone; however, some astrocytes in the ipsilateral striatum and surrounding areas also showed cathepsin X overexpression. On the contrary, no neuronal cells were positive for upregulated cathepsin X in the ipsilateral striatum and throughout other regions following LPS injection. These results are in agreement with our recent *in vivo* study using a hemi-parkinsonian 6-OHDA rat model of PD. Four weeks post-lesion, we observed a persistent cathepsin X upregulation restricted to glial cells concentrated in the ipsilateral SNc (Pislar et al., 2018). The data obtained in the present study are in line also with other reports showing glial upregulation of certain cysteine cathepsins in LPS-induced neuroinflammation (Fan et al., 2012, 2015).

Due to the harmful action of cysteine cathepsins in pathological processes of neurodegeneration, cathepsin inhibitors constitute a possible tool for therapeutic interventions to inhibit excessive proteolytic activity (reviewed in Pislar and Kos, 2014). Some beneficial *in vivo* effects of cysteine protease inhibitors toward neurodegeneration have been demonstrated with cathepsin B inhibition (Hook et al., 2007, 2010; Van Broeck et al., 2007; Haque et al., 2008). The results obtained so far implicate a significant role of cathepsin X in neuroinflammation-induced neurodegeneration; therefore, the inhibition of its excessive activity might be a useful therapeutic approach. Here, we used a known irreversible epoxysuccinyl-based inhibitor of cathepsin X AMS36 (Sadaghiani et al., 2007), which demonstrated promising neuroprotective effects *in vitro* (Pislar et al., 2014, 2017). It has been shown that cathepsin X inhibitor AMS36 significantly reduced LPS-induced production of nitric oxide, reactive oxygen species and the pro-inflammatory cytokines interleukin-6 and tumor necrosis factor-α from microglial BV2 cells. Additionally, inhibition of cathepsin X suppressed microglial activation through the reduced caspase-3 activity, together with diminished microglial cell death and apoptosis, and also through inhibition of the activity of the mitogen-activated protein kinases. In this *in vivo* study, we first demonstrated lower cathepsin X activity in the rat brain after AMS36 administration as well as selectivity for cathepsin X among other cysteine cathepsins. AMS36 selectivity toward cathepsin X was also reported for tumor tissues, whereas in other tissues such as rat liver and kidney, a significant cross-reactivity of AMS36 with cathepsin B inhibition was observed (Sadaghiani et al., 2007). Nevertheless, no decreased activity of cathepsin B has been observed in brain tissue after AMS36 administration. Our first results with AMS36 inhibitor injected *in vivo* indicate its protective effect against LPS-induced neurodegeneration. We have showed reduced extent of LPS-induced striatal degeneration in AMS36 injected rats. Namely, TH levels were no longer significantly reduced after LPS lesion when AMS36 was administred. Similarly, lateral ventricles did not show dilation after LPS and AMS36 injection. Furthermore, the increased striatal iNOS protein level in the ipsilateral striatum following LPS injection compared to the contralateral striatum was abolished when AMS36 was continuously administrated together with LPS injection. These results designate glial cathepsin X as a key player in neuroinflammation-induced neurodegeneration. However, further experiments are needed to unequivocally demonstrate cathepsin X inhibitor induced neuroprotection *in vivo*. Thus, a new generation of selective and reversible cathepsin X inhibitors (Fonovic et al., 2017) may be expected to significantly improve the cathepsin X-targeted therapy of neurodegenerative diseases related with neuroinflammation.

**Figure 8:**
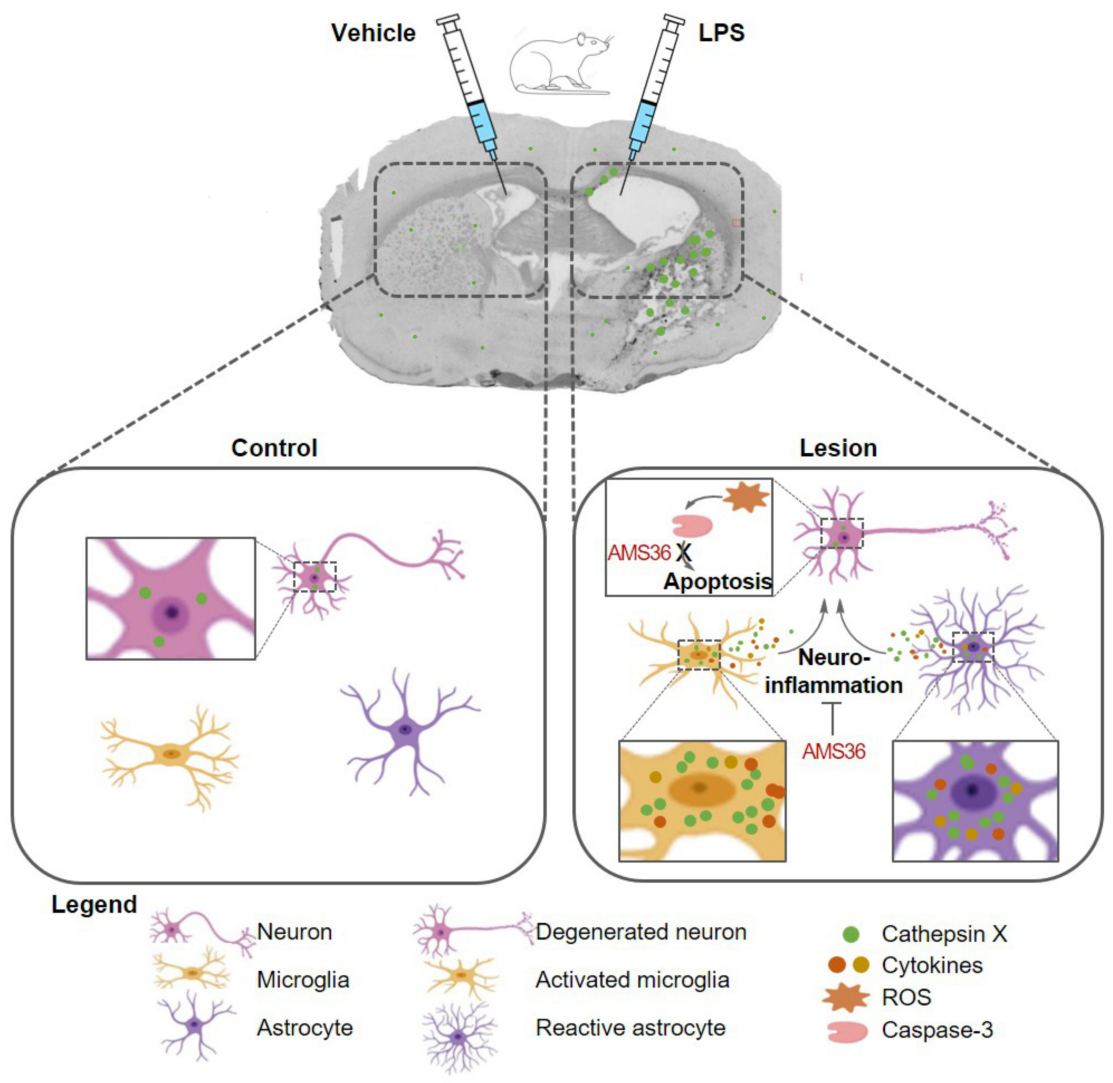
Schematic presentation of glial cathepsin X implication in the striatal neuroinflammation in the LPS model. Unilateral LPS injection into the rat striatum induces cathepsin X upregulation in the striatum and surrounding areas in lesioned hemisphere, predominantyl restricted to activated microglia and reactive astrocytes. Cathepsin X released from glia cells along with secreted cytotokines, could participate in the development of neuroinflammation, which is reflected in the production of reactive oxygen species (ROS) and subsequent caspase cascade activation, namely caspase-3, leading to neuronal apoptosis and death. Inhibition of cathepsin X by small molecule AMS36 potentially ameliorates inflammation-induced neurodegeneration, thus exhibiting neuroprotection role.

In conclusion, in our current study we used an animal model of neuroinflammation by intrastriatal LPS administration to show the regional distribution and cellular localization of cathepsin X, a lysosomal cysteine protease, in the brain. Unilateral LPS injection into the striatum increased cathepsin X expression and its activity in the striatum and surrounding areas on the ipsilateral side. The prominent cathepsin X upregulation was restricted to glial cells, activated microglia and reactive astrocytes. Moreover, administration of a cathepsin X inhibitor along with LPS injection revealed its potential protective role in neuroinflammation induced striatal lesion. As summarized in Figure 8, the obtained results add to previous *in vitro* evidence showing cathepsin X mediated microglia activation and further support the notion that cathepsin X could participate in the development of neuroinflammation in the CNS.

## Data Availability Statement

The original contributions presented in the study are included in the article/Supplementary Materials, further inquiries can be directed to the corresponding author/s.

## Ethics Statement

The animal study was reviewed and approved by Administration of the Republic of Slovenia for Food Safety, Veterinary Sector and Plant Protection, Ljubljana, Slovenia.

## Author Contributions

AP, MŽ, and JK designed the study. AP prepared coronal sections and protein extractions, performed immunohistochemistry staining, Western blotting, ELISA assay, cathepsin X activity assay and double immunofluorescence labeling, generated the data for Figure's and prepared the draft manuscript. LT performed methylene staining and lateral ventricle and striatum area quantification, immunohistochemistry analysis, *in situ* hybridization, generated the data for Figures, participated in carrying out the double immunofluorescence analysis, and reviewed the manuscript. GG reviewed the manuscript. NZ synthesized the cathepsin X inhibitor AMS36. MŽ performed animal surgery. MŽ and JK coordinated the research and reviewed the manuscript. All authors read and approved the final manuscript.

## Conflict of Interest

The authors declare that the research was conducted in the absence of any commercial or financial relationships that could be construed as a potential conflict of interest.

## Abbreviations

6-OHDA: 6-hydroxydopamine
AD: Alzheimer's disease
BSA: bovine serum albumin
Cc: corpus callosum
CNS: central nervous system
Cpu: striatum
Ctx: cortex
DA: dopamine
DMSO: dimethyl sulfoxide
GFAP: glial fibrillary acidic protein
GPe: external globus pallidus
HRP: horseradish peroxidase
iNOS: inducible nitric oxidase synthase
KPBS: potassium phosphate buffer saline
LPS: lipopolysaccharide
NaPBS: sodium phosphate buffer saline
PD: Parkinson's disease
ROS: reactive oxygen species
SNc: substantia nigra compacta
SVZ: subventricular zone
TBST: Tris-buffered saline with 0.1% Tween 20
TH: tyrosine hydroxylase.
