# Supplementary material for: Neuroinflammation-Induced Upregulation of Glial Cathepsin X Expression and Activity in vivo
Source: Front Mol Neurosci. 2020 Nov 20;13:575453. doi: 10.3389/fnmol.2020.575453 (PMC7714997; doi:10.3389/fnmol.2020.575453)
Supplement: Supplementary file 1 [file Data_Sheet_1.docx]

**Neuroinflammation-induced upregulation of glial cathepsin X expression and activity *in vivo***

**Anja Pišlar^1†*^, Larisa Tratnjek^2,3†^, Gordana Glavan^4^, Nace Zidar^5^, Marko Živin^2^, Janko Kos^1,6^**

^1^ Department of Pharmaceutical Biology, Faculty of Pharmacy, University of Ljubljana, Ljubljana, Slovenia

^2^ Institute of Pathophysiology, Medical faculty, University of Ljubljana, Ljubljana, Slovenia

^3^ Institute of Cell Biology, Medical faculty, University of Ljubljana, Ljubljana, Slovenia

^4^ Department of Biology, Biotechnical faculty, University of Ljubljana, Ljubljana, Slovenia

^5^ Department of Pharmaceutical Chemistry, Faculty of Pharmacy, University of Ljubljana, Ljubljana, Slovenia

^6^ Department of Biotechnology, Jožef Stefan Institute, Ljubljana, Slovenia

## Supplementary Figure 1


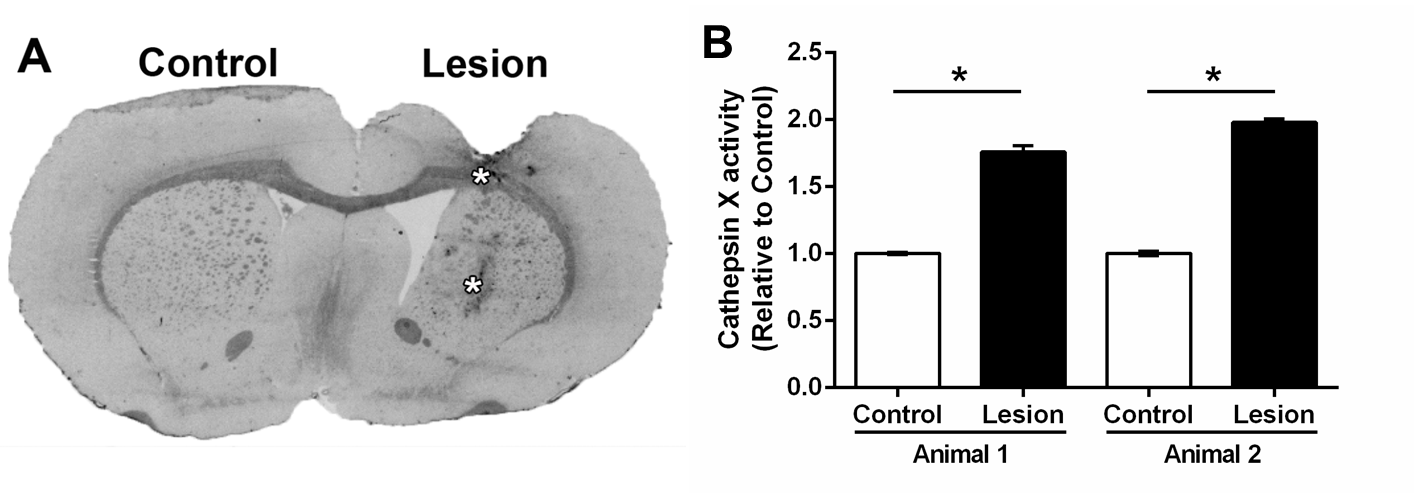


**Supplementary Figure 1**. Upregulation of cathepsin X and its activity in the brains of rats that were administered LPS by unilateral intrastriatal injection (n = 2), but were not treated i.p. with DMSO/saline. (A) Representative cathepsin X immunohistochemical image of the coronal striatal section from the four weeks’ time-point after LPS-induced lesion. Asterisk indicates the areas with increased cathepsin X levels. (B) Enzymatic activity of cathepsin X determined with cathepsin X specific substrate Abz-Phe-Glu-Lys(Dnp)-OH. The analysis of enzymatic activity shows an increase in cathepsin X activity in the lesioned striatum after LPS injection. Results are expressed relative to control and are presented as means ± SD of two independent experiments (n = 2) (two-tailed Student's *t*-test,* *p* ˂ 0.05 vs Control).

## Supplementary Figure 2


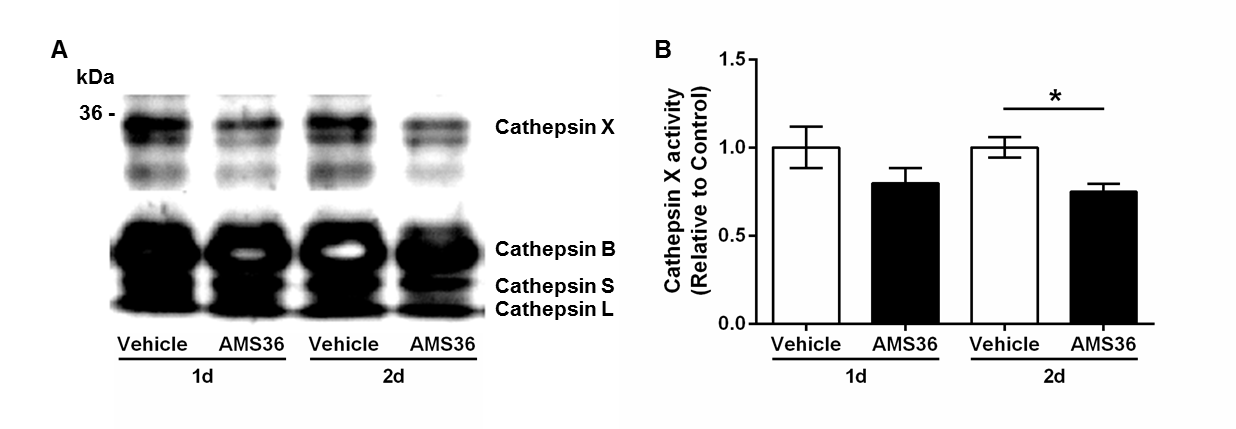


**Supplementary Figure 2.** Assessment of cathepsin X inhibition by cathepsin X inhibitor AMS36 *in vivo*. After 1 and 2 days of the administration of vehicle or inhibitor AMS36 at dose of 50 mg/kg, the cerebellum was removed, homogenized and analyzed for cysteine protease activity. (A) Samples were analyzed by SDS-PAGE, followed by labeling with DCG-04 probe. (B) Enzymatic activity of cathepsin X was determined with cathepsin X specific substrate Abz-Phe-Glu-Lys(Dnp)-OH. Results are expressed relative to control (DMSO) and are presented as means ± SD of three independent experiments (n = 4) (two-tailed Student's *t*-test,* *p* ˂ 0.05 vs Vehicle).

## Supplementary Figure 3


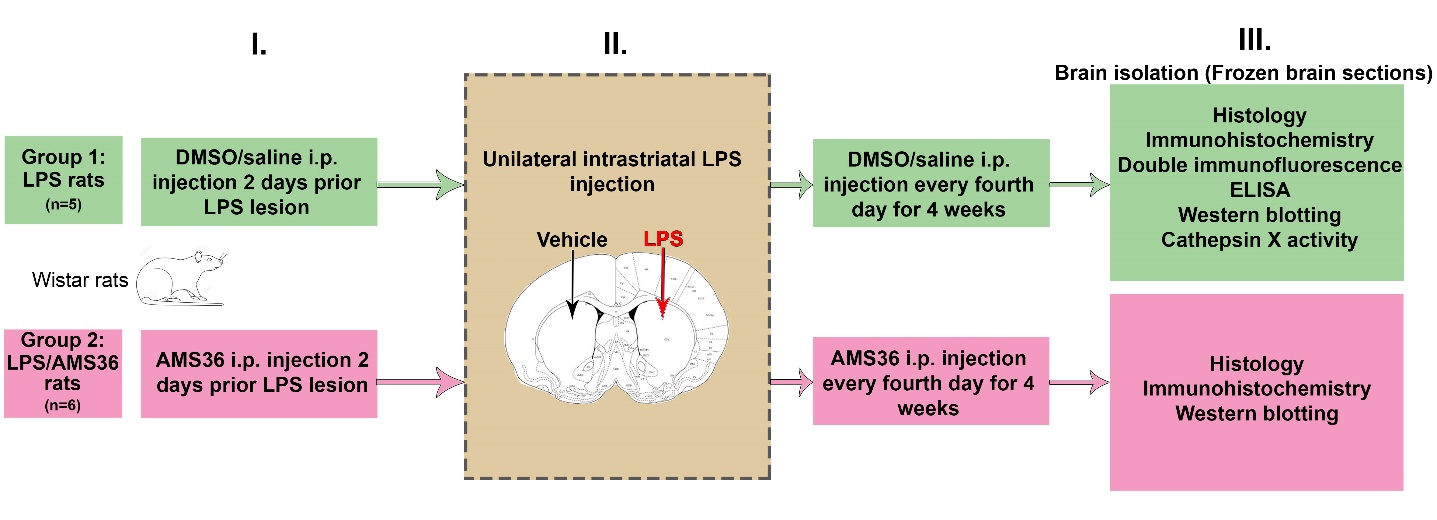


**Supplementary Figure 3.** A graphical outline of the experimental design.

## Supplementary Figure 4


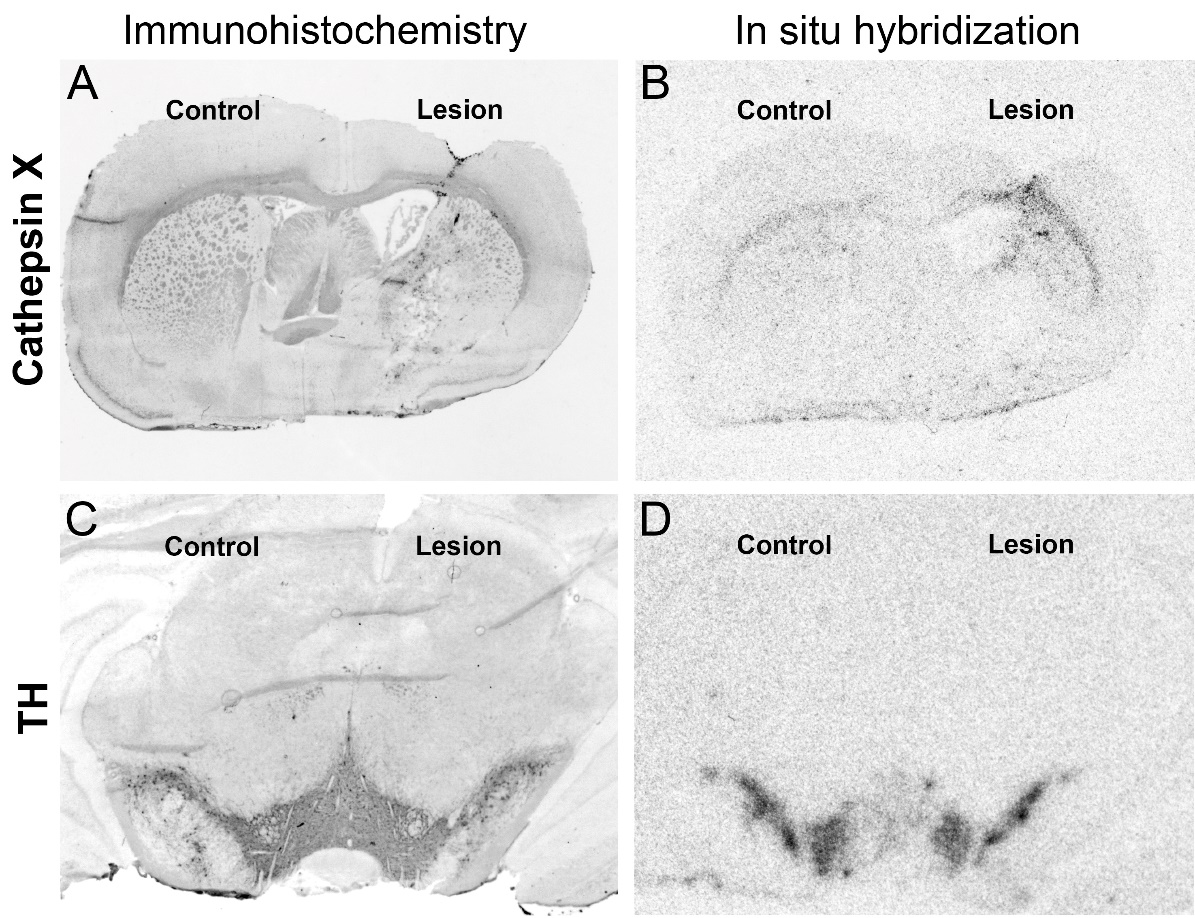


**Supplementary Figure 4.** Cathepsin X protein and mRNA upregulation in the ipsilateral hemisphere after intrastriatal LPS injection. Coronal sections of the striatum (A-B) and SNc (C-D) from the four weeks’ time-point after LPS-induced lesion from the same animal are presented. Cathepsin X immunoreactivity markedly increases in the ipsilateral hemisphere (A) after LPS injection. Similarly, cathepsin X in situ hybridization signal markedly increases in the ipsilateral hemisphere (B) after LPS injection. TH immunostaining and *in situ* hybridization show no loss of TH-positive cells in the SNc.

## Supplementary Figure 5


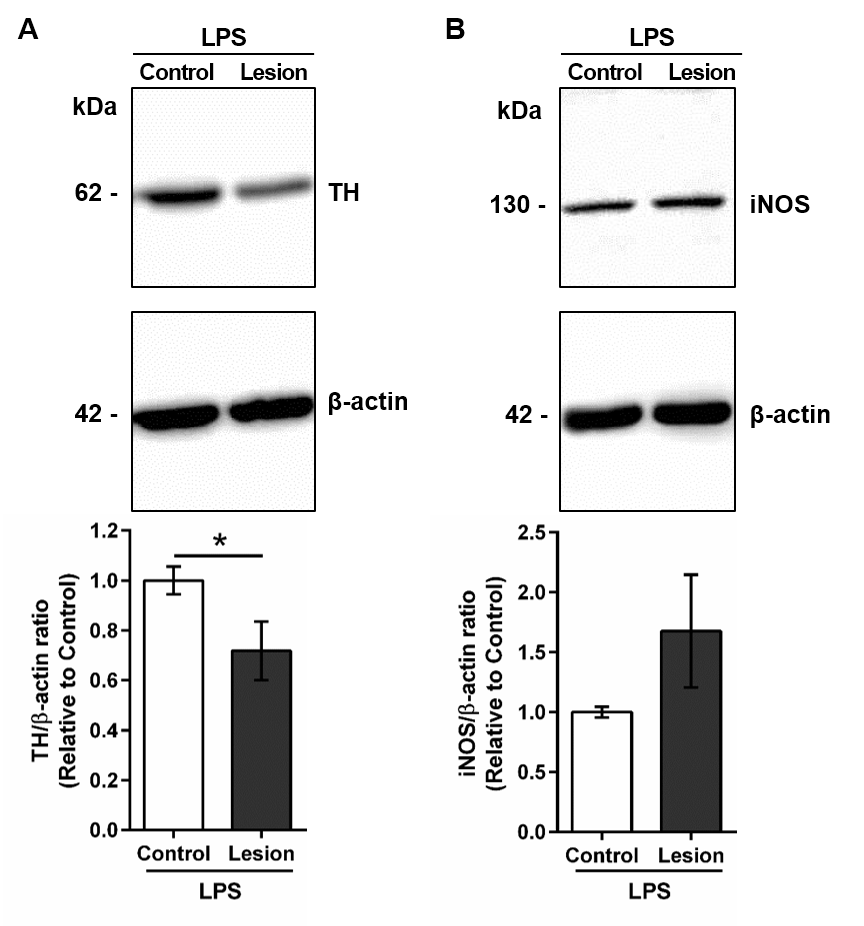


**Supplementary Figure 5.** The protein level of TH and iNOS expression in the striatum after intrastriatal LPS injection. Western blot analysis of TH protein level (A) and iNOS (B) in the contralateral (Control) and ipsilateral (Lesion) dissected striatal sections after four weeks of LPS-induced lesion, using rabbit monoclonal anti-TH antibody and rabbit polyclonal anti-iNOS, respectively. An antibody raised against β-actin was used as loading control. The graphs below blots show a semi-quantitative densitometry analysis of the protein level in the ipsilateral side relative to that in the contralateral side. Values are means ± SD of group of 5 animals (n=5), where 4 anterior-posterior striatal slices of each animal were analyzed (n = 5) (two-tailed Student's t-test, * p ˂ 0.05 vs Control).

## Supplementary Figure 6

**
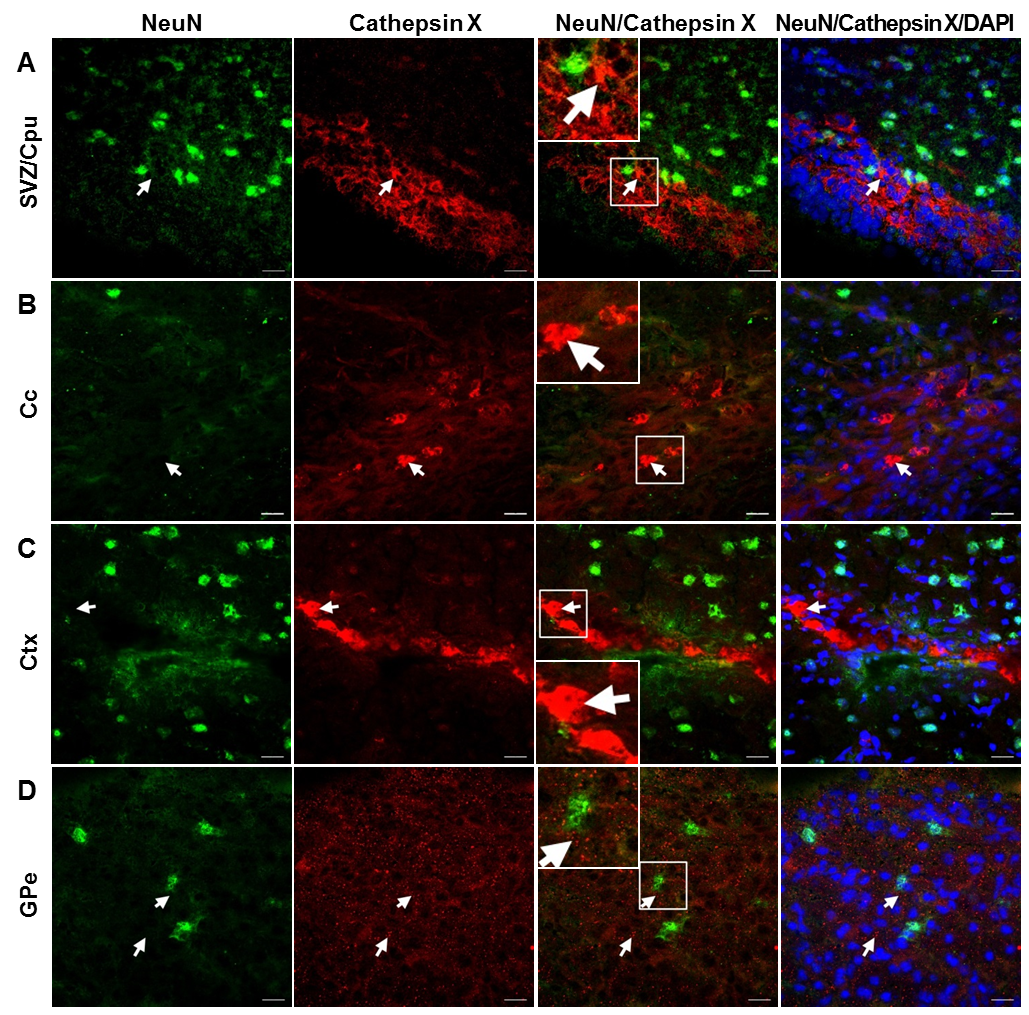
**

**Supplementary Figure 6.** Distribution of cathepsin X in neuronal cells after intrastriatal LPS injection. Representative images of double immunofluorescence staining of neuronal marker NeuN (green fluorescence) and cathepsin X (red fluorescence) in the ipsilateral side of the striatal brain slices four weeks after LPS-induced lesion. Nuclei were counterstained with DAPI (blue fluorescence). In the subventriclular zone/caudate-putamen (SVZ/Cpu), corpus callosum (Cc), cortex (Ctx), and external globus pallidus (GPe) cathepsin X-positive signal was strongly upregulated, however, no neuronal cells were positive for cathepsin X (*arrows*). Group of 5 animals (n = 5) was conducted, where 4 anterior-posterior striatal slices of each animal were analyzed. *Scale bar* = 20 µm.
